# Enhanced Transgene Expression in Sugarcane by Co-Expression of Virus-Encoded RNA Silencing Suppressors

**DOI:** 10.1371/journal.pone.0066046

**Published:** 2013-06-14

**Authors:** San-Ji Gao, Mona B. Damaj, Jong-Won Park, Getu Beyene, Marco T. Buenrostro-Nava, Joe Molina, Xiaofeng Wang, Jessica J. Ciomperlik, Shuga A. Manabayeva, Veria Y. Alvarado, Keerti S. Rathore, Herman B. Scholthof, T. Erik Mirkov

**Affiliations:** 1 Key Laboratory of Sugarcane Biology and Genetic Breeding, Ministry of Agriculture, Fujian Agriculture and Forestry University, Fuzhou, Fujian, China; 2 Department of Plant Pathology and Microbiology, Texas A&M AgriLife Research, Weslaco, Texas, United States of America; 3 *Institute* for International Crop Improvement, Donald Danforth Plant Science Center, Saint Louis, Missouri, United States of America; 4 FCBA-Laboratorio de Biotecnologia, Universidad de Colima, Tecoman, Colima, Mexico; 5 Department of Plant Pathology, Physiology and Weed Science, VirginiaTech University, Blacksburg, Virginia, United States of America; 6 Department of Plant Pathology and Microbiology, Texas A&M University, College Station, Texas, United States of America; 7 National Center for Biotechnology of the Republic of Kazakhstan, Astana, Republic of Kazakhstan; 8 Stoller Enterprises, Inc., Norman E. Borlaug Center for Southern Crop Improvement, Texas A&M University, College Station, Texas, United States of America; 9 Laboratory for Crop Transformation, Institute for Plant Genomics and Biotechnology, Norman E. Borlaug Center for Southern Crop Improvement, Texas A&M University, College Station, Texas, United States of America; University of Leeds, United Kingdom

## Abstract

Post-transcriptional gene silencing is commonly observed in polyploid species and often poses a major limitation to plant improvement via biotechnology. Five plant viral suppressors of RNA silencing were evaluated for their ability to counteract gene silencing and enhance the expression of the Enhanced Yellow Fluorescent Protein (*EYFP*) or the β-glucuronidase (*GUS*) reporter gene in sugarcane, a major sugar and biomass producing polyploid. Functionality of these suppressors was first verified in *Nicotiana benthamiana* and onion epidermal cells, and later tested by transient expression in sugarcane young leaf segments and protoplasts. In young leaf segments co-expressing a suppressor, *EYFP* reached its maximum expression at 48–96 h post-DNA introduction and maintained its peak expression for a longer time compared with that in the absence of a suppressor. Among the five suppressors, *Tomato bushy stunt virus*-encoded P19 and *Barley stripe mosaic virus*-encoded γb were the most efficient. Co-expression with *P19* and *γb* enhanced *EYFP* expression 4.6-fold and 3.6-fold in young leaf segments, and GUS activity 2.3-fold and 2.4-fold in protoplasts compared with those in the absence of a suppressor, respectively. In transgenic sugarcane, co-expression of *GUS* and *P19* suppressor showed the highest accumulation of GUS levels with an average of 2.7-fold more than when *GUS* was expressed alone, with no detrimental phenotypic effects. The two established transient expression assays, based on young leaf segments and protoplasts, and confirmed by stable transgene expression, offer a rapid versatile system to verify the efficiency of RNA silencing suppressors that proved to be valuable in enhancing and stabilizing transgene expression in sugarcane.

## Introduction

RNA silencing is an ancient pathway shared by eukaryotic organisms to regulate gene expression. It particularly operates as an adaptive defense mechanism, which is initiated by the formation of double stranded RNAs (dsRNAs) to destroy aberrant RNAs in the cell [1]–[4]. The silencing pathway is very complex in higher eukaryotes, but some of its distinct steps and key components are well characterized. The dsRNA trigger is first cleaved by the RNase III-type DICER-LIKE proteins into small RNA species of 21–26 nucleotide duplexes named short-interfering RNAs (siRNAs) or microRNAs (miRNAs) [5], [6], which are denatured and incorporated into the multi-component RNA-induced silencing complex (RISC) with an Argonaute (AGO) protein at its catalytic core [7]. The RISC complex then binds complementary mRNAs guided by single-stranded siRNAs, thereby mediating processes such as translational inhibition, RNA degradation or chromosome modification [8]–[10]. Unlike the miRNAs produced by the miRNA precursors [5], [6], [10], the siRNAs can also be amplified from the target RNA by cellular host RNA-dependent RNA polymerases (RdRPs) to produce additional dsRNAs that will be processed into secondary siRNAs [11]. The Suppressor of Gene Silencing 3, a dsRNA binding protein, is also required for post-transcriptional gene silencing (PTGS) in plants [12], [13].

Plant viruses have evolved several counter-defensive strategies to efficiently suppress their host RNA silencing mechanism. The production of virus-encoded suppressors of RNA silencing is one of the strategies used to counteract host antiviral defense [14]–[16]. So far, several suppressors of RNA silencing have been identified from different types of viruses, and they show a high diversity in primary sequence and protein structure, though sharing certain mechanistic features [16]–[18]. Viral suppressors seem to interfere with the RNA silencing pathway at distinct steps, since they potentially have different molecular targets in the host and operate differently in widely used silencing inhibition assays [19], [20]. For instance, the *Tomato bushy stunt virus* (TBSV)-encoded P19 [21], [22], one of the most studied suppressors, sequesters 21-nt siRNAs in a non-specific manner, preventing their incorporation into the RISC complex to act as guides; it also inhibits the spread of the ds siRNA duplex identified as the signal of RNA silencing [18], [23] as well as the translational efficiency of AGO1 mRNA by modulating the endogenous miR168 level [24]. The suppression activity of the *Barley stripe mosaic virus* (BSMV)-encoded γb was demonstrated in *Agrobacterium*-mediated transient assays [25], [26], and the molecular basis of its silencing suppression is similar to that of P19 [20].

The P1/HC-Pro or HC-Pro from *Potato virus Y* [27] or *Tobacco etch virus* (TEV) [28] was the first identified suppressor serving as a model to study the mechanism of silencing suppression. HC-Pro is proposed to act on the RISC complex [20], [29] or downstream of an RdRP by interfering with the DICER protein [30], [31], or by sequestrating the 21-nt siRNA duplexes [19], [20] and inhibiting the 3′ modification of the si/miRNAs [29], [32].

Transgenic and transient expression via *Agrobacterium* co-infiltration into *Nicotiana benthamiana* and *Arabidopsis thaliana* have been extensively used to probe the phenomenon of RNA silencing and the function of viral suppressors at the whole plant level [17]. Protoplasts of *N. benthamiana* and *A. thaliana* proved useful to investigate transient gene expression [33], RNAi-mediated silencing of gene expression [34], [35] and the RNA silencing suppressor function [36]–[38] at the cellular level. Viral suppressors of silencing were quantitatively evaluated by transient co-expression with the *Green Fluorescent Protein* (*GFP*) in germinating lima bean (*Phaseolus lunatus* L.) cotyledons via particle bombardment [39].

Sugarcane (*Saccharum* spp. hybrid) is an economically important sugar and bioenergy producing polyploid crop, which is amenable for improvement through genetic engineering [40]–[42]. Transgene silencing is currently one of the major limiting factors to produce improved transgenic varieties, and to achieve commercially useful expression levels of transgenes in this crop [43]–[45]. In the present study, the strategy of using viral RNA silencing suppressors to counteract RNA silencing was adopted in sugarcane in an attempt to enhance transgene expression and stability. Four RNA silencing suppressors were evaluated for their silencing suppression efficiency by their transient and stable transgenic co-expression with the Enhanced Yellow Fluorescent Protein (*EYFP*) or the β-glucuronidase (*GUS*) reporter gene. These include the TEV-encoded P1/HC-Pro, the BSMV-encoded γb, the TBSV-encoded P19, and one putative suppressor, the *Sugarcane bacilliform virus* (SCBV)-encoded OrfΙ. In addition, a P19 suppressor mutant, P19/R43W, whose overexpression did not induce developmental defects, was also evaluated [46]. The suppressor-reporter gene constructs were tested in the dicot *Nicotiana benthamiana* plants and monocot onion epidermal cells, to determine that they are expressing functional suppressors. An efficient transient suppressor-reporter gene co-expression system, based on young leaf segments and protoplasts of sugarcane, was first established and it was subsequently used to demonstrate that several silencing suppressors enhanced *EYFP* and *GUS* expression to a significant level. That the transient expression system provided a rapid analysis of viral RNA silencing suppressor efficiencies was further supported by the generation of stable transgenics. Combined, these results show the usefulness of the system to probe the activity of these suppressors, while these proved to be valuable in enhancing and stabilizing transgene expression in sugarcane.

## Results

### Assaying the Activity of Viral RNA Silencing Suppressors in Model Plant Systems

To verify that the genetic constructs were expressing functional suppressors (P1/HC-Pro, P19, and γb), we first tested these in *N. benthamiana* using the standard suppressor activity assay [21] (Legend, Figure S1). A construct expressing *GFP* was co-agroinfiltrated with a suppressor-expressing construct into *N. benthamiana* leaves, and *GFP* expression was compared to the treatment with GFP construct alone. These experiments verified that expression of the suppressors led to the expected enhanced and prolonged *GFP* expression in the dicot *N. benthamiana* (Figure S1).

To determine whether the suppressors were active in a monocot system, onion epidermal cells were co-bombarded with constructs expressing the *EYFP* gene and those expressing the suppressor genes (Figure S2a). In this case, the SCVB OrfI suppressor was also included. It has been established that an increase in the number of fluorescent cells correlates with the effectiveness of suppressor activity [47], thus we monitored the effect of co-bombardment with a suppressor on the number of *EYFP*-expressing cells (Figure S2b). The results of these comparisons indicated that, unlike in *N. benthamiana*, for unknown reasons not all suppressors performed optimally, even though the suppression effect was often more evident when two suppressors were combined (Figure S2b). For instance, the SCVB OrfI and γb suppressors exhibited the most prominent effect (Figure S2b), but whether this is related to their origin of being a monocot-infecting virus remains to be determined. Due to the relative small number of cells that expressed *EYFP* (Figure S2a), quantification with western analysis was technically not feasible, so any suppressor effect at the cellular level could not be quantified. Therefore, either the onion cells yielded unexpected results in not responding to certain suppressors in an expected manner to be explored in future experiments, and/or the system itself was insufficiently quantifiable. Thus, we felt that a more robust transient system for monocot expression, preferably sugarcane itself, needed to be established.

### Development of Transient Expression Systems for the Rapid Testing of viral RNA Silencing Suppressor Efficiencies in Sugarcane

To evaluate the ability of viral RNA silencing suppressors in enhancing transgene expression in sugarcane, we first established two rapid and efficient transient systems for the co-expression of the suppressor and the reporter gene in the same tissues or cells.

#### Optimization of transient expression in vivo in young leaf segments

To investigate the optimal parameters of imaging *EYFP*-expressing cells in sugarcane young leaf segments following bombardment, we used multicolor fluorescence imaging at low and high optical magnifications combined with ImageJ data analysis. Images of young leaf segment cells were acquired at 48 h after bombardment with pUbi:*EYFP*:Tnos (*EYFP* under the control of the maize *ubiquitin 1* (Ubi) promoter and the *Agrobacterium* nopaline synthase terminator (Tnos); Figure S3) (0.5 µg) using filter sets for different fluorophores such as rhodamine, eGFP and YFP with different detection spectra, at 15x and 150x magnifications. Imaging at a high magnification (150x) allowed the distinction of *EYFP*-expressing cells from autofluorescence derived from damaged cells or any object present on the tissue surface, but the field of vision was restricted to few cells only; however, imaging at a low magnification (15x) provided a significantly larger area of cells for image capture and analysis (Figures S4 and S5). The overlaid image generated from merging the YFP and bright field images clearly showed that the *EYFP*-expressing cells were intact (Figure S5). Furthermore, images taken with the rhodamine filter failed to show any prominent fluorescent spots, indicating that there is no visible autofluorescence from damaged/dead cells in leaf segments expressing pUbi:*EYFP*:Tnos+pUbi:Tnos or from those bombarded with pUbi:Tnos (Figure S3) or water (negative controls) at 15x and 150x magnifications (Figures S4 and S5). Quantitative assessment of *EYFP* expression in leaf segments of negative controls using ImageJ indicated that the level of autofluorescence from damaged/dead cells or any object present on the tissue surface is minor (Figure S6). Although the *EYFP* foci count number of both negative controls (12.10±0.30) was about 9.4% of the total foci count number of pUbi:*EYFP*:Tnos+pUbi:Tnos (129.40±15.70), their *EYFP* expression level (gray value×pixels) (0.01×10^5^) was only 1% of that of pUbi:*EYFP*:Tnos+pUbi:Tnos (0.95×10^5^±0.15×10^5^) (Figure S6). Since the degree of cell damage during bombardment and the chance of having foreign objects on the tissue surface will be the same for each treatment, the level of autofluorescence derived from damaged/dead cells or any foreign object present on the tissue surface can be assumed to be uniform for all treatments. Therefore, we are confident that the sugarcane leaf segment- based transient gene expression system and the data analysis by ImageJ coupled with fluorescent microscopy at 15x magnification, adopted in the current study, provide a reliable approach for the quantitative analysis of *EYFP* expression in order to investigate the effect of RNA silencing suppressors in sugarcane.

To optimize the conditions of transient expression of *EYFP* in sugarcane young leaf segments, we investigated the duration of 0, 3 and 5 days of pre-culture of leaf segments on media prior to bombardment and the dosage of the introduced gene. Data collected at 48 h after bombardment with *EYFP* (pUbi:*EYFP*:Tnos; Figure S3) showed that leaf segments bombarded without pre-culture (0 day) had the lowest *EYFP* expression (expression level: 3.65×10^5^±0.26×10^5^; foci count: 228.00±17.00). However, leaf segments pre-cultured for 3 days and 5 days displayed significantly (*p*<0.05) higher *EYFP* expression levels (8.12×10^5^±0.62×10^5^ and 7.41×10^5^±0.56×10^5^, respectively) and foci counts (438.00±16.00 and 443.00±27.00, respectively) than those without pre-culture at 48 h after bombardment. These results show that pre-culture of sugarcane young leaf segments for 3–5 days prior to bombardment enhanced *EYFP* expression (as measured by *EYFP* foci count and expression level).

To determine a suitable amount of DNA to be used for a higher *EYFP* expression, six *EYFP* plasmid (pUbi:*EYFP*:Tnos) concentrations (from 0.125 µg to 4.0 µg DNA per bombardment) were tested in 3 day-pre-cultured sugarcane young leaf segments. Data collected at 48 h after bombardment showed that *EYFP* expression increased in a linear manner with increasing amounts of *EYFP* DNA from 0.125 µg (foci count: 237.00±18.00; expression level: 1.19×10^5^±0.10×10^5^), to 0.25 µg (262.00±14.00; 2.70×10^5^±0.10×10^5^) and to 0.5 µg (320.00±17.00; 4.80×10^5^±0.40×10^5^), until it reached a plateau with 1.0 µg, 2.0 µg and 4.0 µg. The highest *EYFP* foci count of 369.00 (±29.00) and expression level of 6.70×10^5^ (±0.70×10^5^) were obtained with 4 µg of *EYFP* DNA per bombardment, but this increase was not significantly different from the one obtained with 0.5 µg, 1 µg or 2 µg of *EYFP* DNA per bombardment. However, we opted to use the 0.25 µg dose in the transient experiments with young leaf segments, to decrease the fluorescence background of *EYFP* expression (from the *EYFP* plasmid alone) and avoid interference with image data collection (data not shown).

#### Establishment of a cellular transient expression system based on protoplasts

A homogenous cell suspension was obtained from compact globular white-yellow embryogenic sugarcane callus, originating from leaf rolls (Figures S7a–b), in liquid MS medium with 2,4-dichlorophenoxyacetic acid (2,4-D) (3 mg/L). Subsequently, protoplasts were successfully isolated from this cell suspension after culturing for 2–3 days, with an average yield of about 2×10^6^ protoplasts per 1 mL of suspension (Figure S7c). The polyethylene glycol (PEG)-calcium chloride (CaCl_2_) transfection efficiency of the isolated protoplasts (1×10^5^ protoplasts; 100 µL) was assessed by using 10 µg of *EYFP* plasmid DNA (pUbi:*EYFP*:Tnos) and three transfection (protoplast incubation with DNA) periods of 5 min, 10 min and 15 min. Image analysis data collected at 24 h after co-transfection of the protoplasts showed that around 30% of the transfected protoplasts expressed *EYFP,* i.e. 30% of protoplasts were successfully transfected (Figures S7d–f). This transfection rate was maintained to the same level during the 15 min time period (data not shown), indicating that this is a less critical factor to be considered.

#### Dosage effect of the viral RNA silencing suppressors on transgene expression

**Figure 1:**
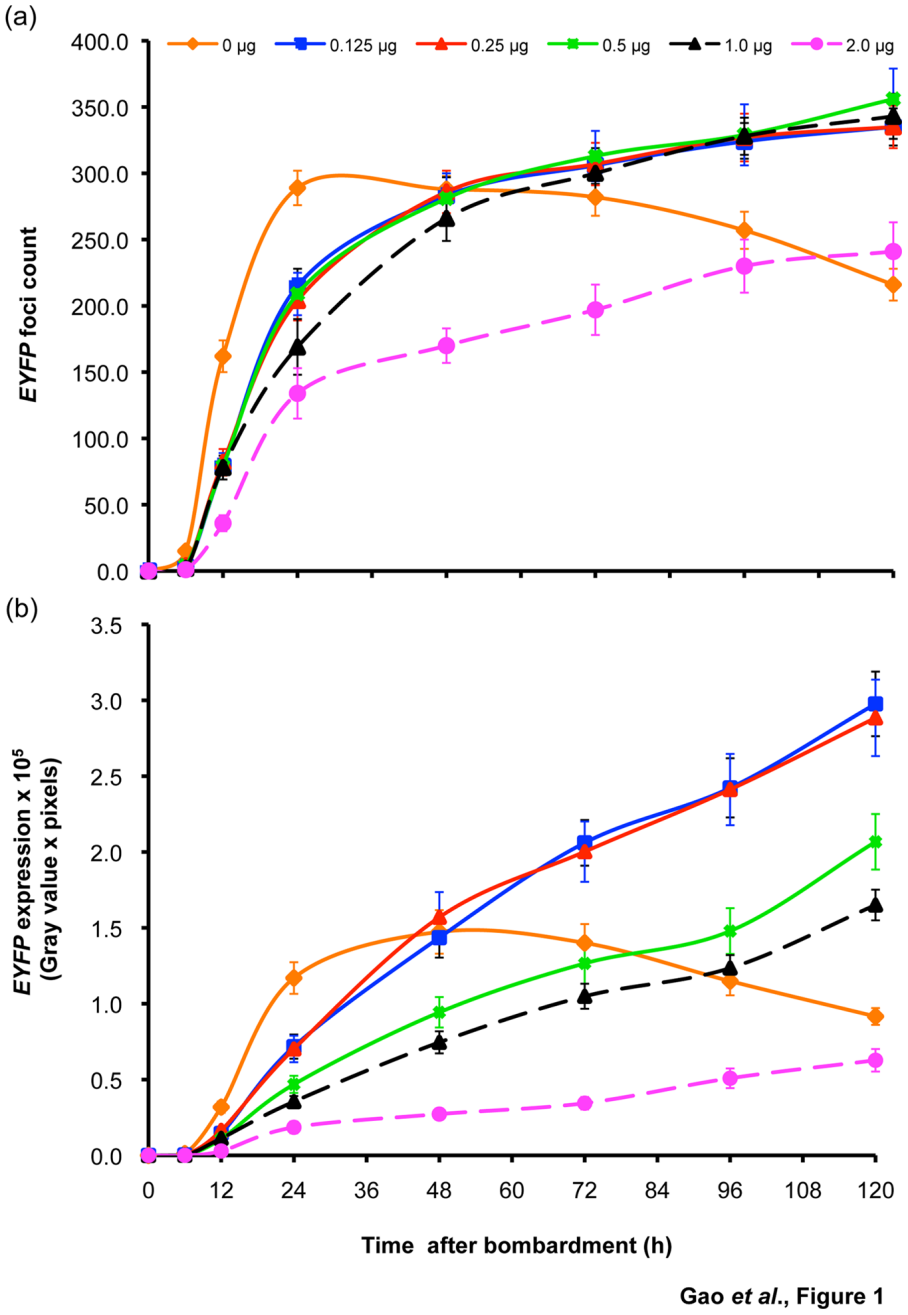
Dosage effect of the TBSV-encoded P19 RNA silencing suppressor on transient expression of the *EYFP* reporter gene in sugarcane young leaf segments. *EYFP* (pUbi:*EYFP*:Tnos; Figure S3) (0.25 µg per shot) was co-bombarded with each of five concentrations of *P19* DNA (pUbi:*P19*:Tnos; Figure S3), and *EYFP* expression as measured by foci count (a) and expression level (b) was monitored for 120 h post-bombardment of sugarcane young leaf segments (3 day-pre-cultured). Vector with no suppressor (pUbi:Tnos; Figure S3) was used as a negative control. Values represent two independent experiments and ten technical repeats, and are reported with the standard error. Quantitation of *EYFP* foci counts and expression levels is provided in Materials and Methods.

Because P19 enhanced transgene expression in *N. benthamiana* and in our preliminary experiments using sugarcane young leaf segments, we determined the optimal amount of the *P19* suppressor to increase *EYFP* expression and GUS activity in sugarcane young leaf segments and protoplasts, respectively. When *EYFP* (pUbi:*EYFP*:Tnos) (0.25 µg) was introduced with no suppressor in 3 day-pre-cultured young leaf segments, *EYFP* expression reached its peak at 24 h (*EYFP* foci count) or 48 h (*EYFP* expression level) post-introduction and then declined (Figures 1a and b). However, co-bombardment of *EYFP* (0.25 µg) with increasing doses of *P19* (pUbi:*P19*:Tnos; Figure S3) extended the *EYFP* expression peak to at least 72 h and enhanced *EYFP* expression (foci count and expression level) (Figures 1a and b). The highest increases in *EYFP* expression were obtained with 0.125 µg (335.00±14.00; 2.90×10^5^±0.30×10^5^) and 0.25 µg (335.00±16.00; 2.10×10^5^±0.20×10^5^) of *P19.* The 0.125 µg and 0.25 µg *P19* concentrations resulted in 1.6-fold increase in *EYFP* foci counts, and 3.2-fold and 2.3-fold increase in *EYFP* expression levels, respectively, compared to those in the absence of *P19* (216.00±12.00; 0.90×10^5^±0.10×10^5^) at the 120 h time point (Figures 1a and 1b). Co-introduction of *EYFP* (0.25 µg) with higher doses of *P19,* such as 0.5 µg and 1.0 µg, still enhanced the *EYFP* foci count (356.00±23.00 and 343.00±17.00 at 120 h, respectively) by 1.7-fold and 1.6-fold, respectively, but a significant decrease was observed with the 2-µg dose (241.00±22.00 at 120 h) (Figure 1a).

Similarly, increasing the amount of the *P19* suppressor co-transfected with *GUS* was observed to enhance GUS activity in sugarcane protoplasts (Table 1). Co-expression of protoplasts (1×10^5^) with *GUS* (pUbi:*GUS*:Tnos, Figure S3) (5 µg) and *P19* (pUbi:*P19*:Tnos) at 2.5 µg (61.80±3.50) and 5.0 µg (89.20±5.60) resulted in a significant (*p*<0.05) increase of 1.6-fold and 2.4-fold more than those to those transfected with *EYFP* with no suppressor (vector) (37.60±3.20), respectively (Table 1). The highest level of GUS activity was reached with 10 µg of *P19* (96.80±5.00) (Table 1).

**Table 1:** Dosage effect of the TBSV-encoded P19 RNA silencing suppressor on transient expression of the *GUS* reporter gene in sugarcane protoplasts.

P19 DNA(µg)	GUS activity(pmoles of 4-methylumbelliferone/min/µg protein)
**Vector-no P19**	37.60±3.20 c
**0.0- sterile water**	31.60±1.80 c
**2.5**	61.80±3.50 b
**5.0**	89.20±5.60 a
**10.0**	96.80±5.00 a

DNA (5 µg) from pUbi:*GUS*:Tnos (Figure S3) was co-transfected into a protoplast suspension (100 µL; 1×10^5^ protoplasts) with three concentrations of *P19* DNA (pUbi:*P19*:Tnos) (Figure S3), respectively, and GUS activity of protoplasts was measured at 24 h post-transfection. Vector with no *P19* suppressor (pUbi:Tnos; Figure S3) and sterile water were used as controls. Values represent three biological samples and six technical repeats, and are reported with the standard error. Means with the same letter are not significantly different (*p*>0.05).

### Enhancement of Transient Gene Expression by Co-expression of viral RNA Silencing Suppressors in Sugarcane Young Leaf Segments and Protoplasts

**Figure 2:**
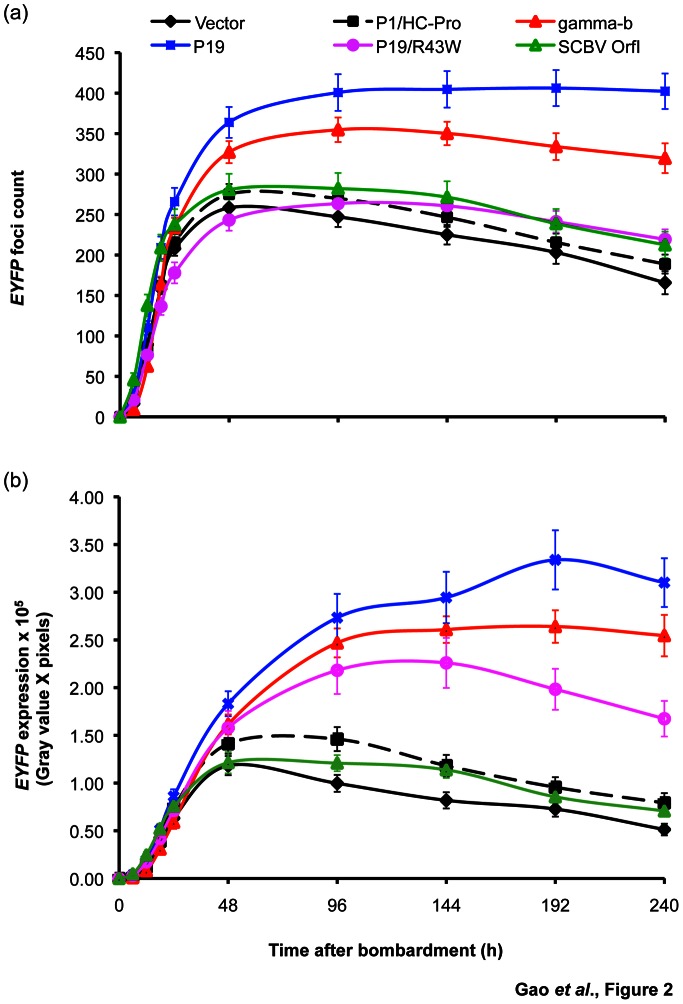
Quantitative assessment of the effect of viral RNA silencing suppressors on transient expression of the *EYFP* reporter gene in sugarcane young leaf segments. *EYFP* expression as measured by foci count (a) and expression level (b) was monitored in 3 day-pre-cultured young leaf segments for 240 h after co-bombardment with 0.25 µg (per shot) of *EYFP* (pUbi:*EYFP*:Tnos; Figure S3) and 0.5 µg (per shot) each of RNA silencing suppressors (under the control of the Ubi promoter and nos terminator; Figure S3), *P1/HC-Pro*, *γb*, *P19*, *P19/R43W* and *SCBV OrfI*. Vector with no suppressor (pUbi:Tnos; Figure S3) was used as a negative control. Values represent means with standard error from three independent experiments and 8–10 replicates per experiment. Quantitation of *EYFP* foci counts and expression levels is provided in Materials and Methods. gamma-b: γb.

After establishing the two transient expression systems for sugarcane, we tested the five suppressors, P19, P19/R43W, γb, P1/HC-Pro and SCBV OrfI (under the control of the Ubi promoter and nos terminator; Figure S3), in young leaf segments and in protoplasts. In the leaf segment system, when *EYFP* (pUbi:*EYFP*:Tnos) was introduced alone, *EYFP* expression peaked at 48 h post-bombardment (foci count: 258.00±11.00; expression level: 1.20×10^5^±0.10×10^5^) and then declined rapidly (Figure 2a and 2b; Figure S8). However, when *EYFP* was co-introduced with one of the five suppressors, *EYFP* expression reached its maximum within 48–96 h post-bombardment and maintained its peak for a longer time (Figures 2 and S8). P19 and γb induced the highest peaks of *EYFP* expression levels (3.30×10^5^±0.30×10^5^ and 2.60×10^5^±0.20×10^5^, respectively) and foci counts (406.00±22.00 and 334.00±17.00, respectively) at 192 h post-bombardment (Figures 2a and 2b). Each of these two suppressors resulted in a highly significant improvement in the transient transformation efficiency, as shown by up to a 4.6-fold and 3.6-fold increase in the *EYFP* expression level at 192 h after bombardment, respectively, when compared to the control (absence of a suppressor) (expression level: 0.70×10^5^±0.10×10^5^) (Figure 2b). P19/R43W (a P19 mutant) also enhanced *EYFP* expression to significant levels (2.30×10^5^±0.30×10^5^), i.e. 2.8-fold increase in *EYFP* expression level compared to that with *EYFP* in the absence of a suppressor (0.80×10^5^±0.10×10^5^) at 144 h post-bombardment (Figure 2b). P1/HC-Pro and SCBV OrfΙ showed no significant effect (p>0.05) on *EYFP* expression (Figures 2 and S8).

**Figure 3:**
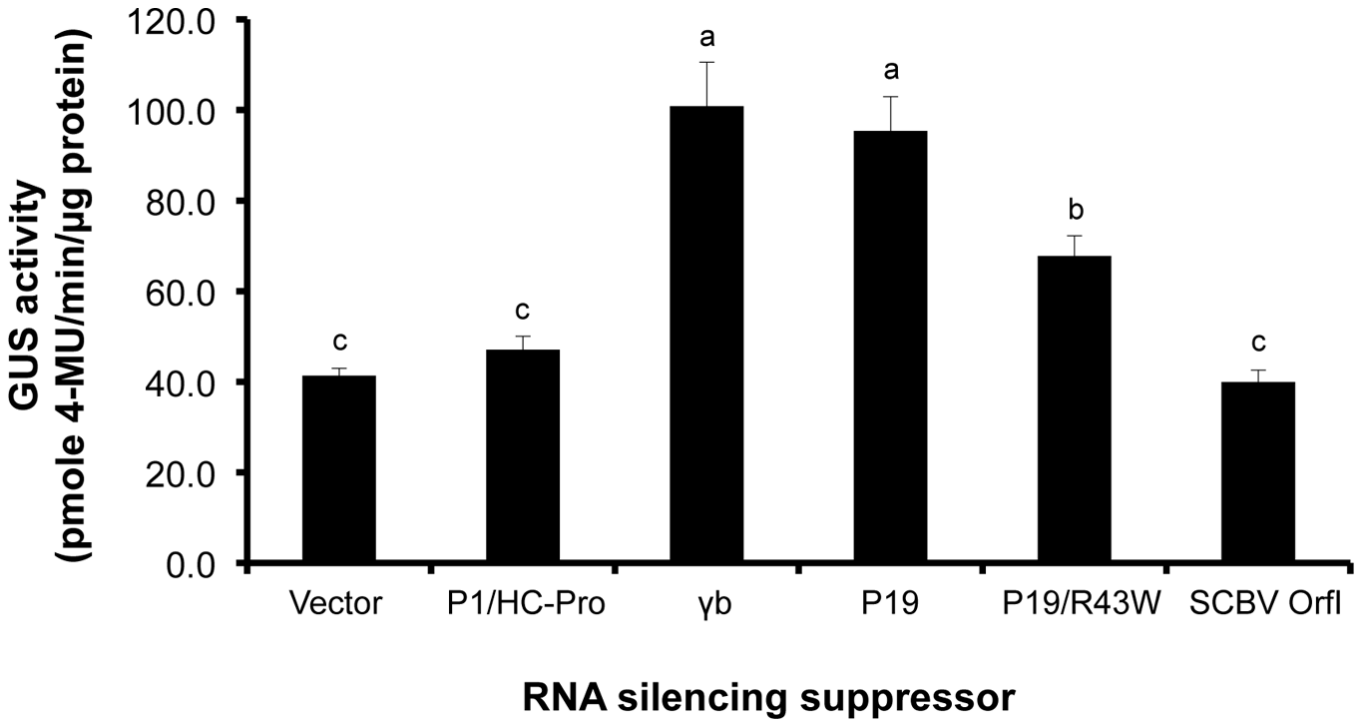
Quantitative assessment of the effect of viral RNA silencing suppressors on transient expression the *GUS* reporter gene in sugarcane protoplasts. The GUS activity of protoplasts was monitored for 24 h after co-transfection of protoplasts (1×10^5^ protoplasts; 100 µL) with 5 µg of *GUS* (pUbi:*GUS*:Tnos; Figure S3) and 10 µg each of RNA silencing suppressors (under the control of the Ubi promoter and nos terminator; Figure S3), *P1/HC-Pro*, *γb*, *P19*, *P19/R43W* and *SCBV OrfI*. Vector with no suppressor (pUbi:Tnos; Figure S3) was used as a negative control. Values represent means with standard error from three independent experiments and six replicates per experiment. Means with the same letter are not significantly different (*p*>0.05).

In the protoplast system, we quantified expression of *GUS* at 24 h after co-transfection with or without a suppressor. When co-expressed with *GUS*, each of *γb* (100.84±41.17), *P19* (95.40±32.00), and *P19/R43W* (67.78±18.82) enhanced GUS activity by 2.4-fold, 2.3-fold and 1.6-fold, respectively, as compared to *GUS* in the absence of a suppressor (41.33±6.91) (Figure 3). Co-transfection of protoplasts with *GUS* and each of *P1/HC-Pro* and *SCBV OrfΙ* resulted in no significant increase in GUS activity (Figure 3).

### Enhancement of Stable Transgene Expression by Co-expression of the Viral RNA Silencing Suppressor P19 in Sugarcane

**Figure 4:**
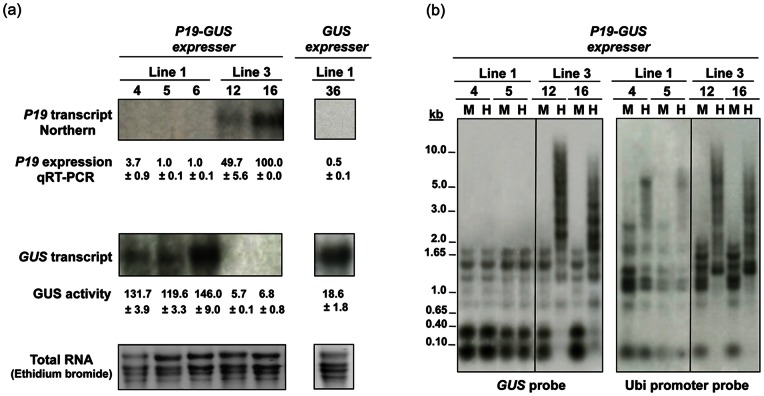
Enhanced expression of the *GUS* reporter gene by stable co-expression of the TBSV-encoded *P19* suppressor in transgenic sugarcane. (a) Relative abundance of *P19* and *GUS* transcripts was determined by northern blot and quantitative RT-PCR (qRT-PCR) analyses in two representative *P19-GUS* transgenic sugarcane lines co-expressing *GUS* and *P19* (two plants per line). Lines expressing *GUS* with no suppressor were used as a control. Blots of RNA (15 µg per sample) were probed with radioactively labeled *P19* DNA, stripped and then reprobed with *GUS* DNA. Normalized qRT-PCR *P19* expression levels of the *P19-GUS* lines are reported as a percentage, relative to that of the highest expressing plant. GUS activity (pmoles of 4-methylumbelliferone/min/µg protein) of the *P19-GUS* lines is also indicated. Values represent three biological samples and three technical repeats, and are reported with the standard error. (b) Methylation status of the coding region and promoter of the *GUS* reporter gene in the *P19-GUS* transgenic sugarcane lines. Southern blot of genomic DNA (10 µg per sample) of two representative *P19-GUS* lines, one non-silenced (Line 1, plants 4 and 5) and one silenced (Line 3, plants 12 and 16), digested with methylation-sensitive *Hpa*II (H), and methylation-insensitive *Msp*I (M), restriction endonucleases, were probed with the *GUS* gene or the Ubi promoter. Shifts in DNA hybridization fragments indicate methylation.

In order to study the long term protection of transgene expression by the RNA silencing suppressor P19, a total of 41 transgenic plants, representing seven independent stably transformed lines were generated, from leaf roll disc explants, by co-bombardment of the *GUS* reporter (pUbi:*GUS*:Tnos) and the *P19* (pUbi:*P19*:Tnos) suppressor genes. Successful gene co-integration was confirmed by Southern blot analysis (data not shown). Lines co-expressing *P19* and *GUS (P19-GUS)* developed normally and showed a significant (*p*<0.05) enhancement in GUS activity, i.e. an average of 53.2% increase when compared to lines expressing *GUS* alone (Table 2; Figure 4a). Significant (*p*<0.05) increases of 1.9-fold (in 39% of transgenics) to 3.5-fold (in 20% of transgenics) in GUS activity were observed in *P19-GUS* transgenics when compared to those expressing *GUS* alone (Table 2). No significant increase in GUS activity was noted in the remaining of the *P19-GUS* transgenics (16 plants) (data not shown).

**Table 2:** Effect of the TBSV-encoded P19 RNA silencing suppressors on the expression of the *GUS* reporter transgene in transgenic sugarcane.

Transgenic	GUS activity (pmoles of 4-methylumbelliferone/min/µg protein)
***P19-GUS***	
20%	129.90±5.90 a (119.60–146.00)
39%	70.10±5.00 b (53.80–98.90)
***GUS***	37.00±4.10 c (18.60–63.30)
**Non-transgenic**	5.70±0.90 d (3.80–6.70)

Average GUS activity was measured in leaves of 4-month-old sugarcane transgenic lines co-expressing pUbi:*GUS* and pUbi:*P19* (7 lines; 41 plants analyzed). pUbi:*GUS* transgenic (3 lines; 5 plants analyzed) and non-transgenic sugarcane (3 plants) were included as controls. For each set of experiments, the range of GUS activity values is indicated in parenthesis. Values represent three biological samples and three technical repeats, and are reported with the standard error. Values with the same letter are not significantly different (*p*>0.05). 20% and 39% represent the percentage of plants that are transgenic for the *P19* and *GUS* genes.

P19 expression and protein accumulation in the high *GUS* expressing *P19-GUS* plants (belonging to 6 lines) were below detectable levels (Northern and qRT-PCR, Figure 4a; western data not shown). This phenomenon has been previously reported in different species transformed with the wild-type *P19* gene [31], [48]–[51]. However, *P19* was highly expressed in the low *GUS* expressing *P19-GUS* plants (one line) (Figure 4a).

Even though seven independent transgenic lines were generated, a detailed molecular analysis is presented here for the representative highest *GUS* expressing line 1 and the lowest *GUS* expressing line 3. For instance, *P19* was expressed in *P19-GUS* line 1 (three representative plants) by an average of only 1.9% relative to the *P19* highest expressing plant (100%) of *P19-GUS* line 3 (Figure 4a). GUS levels, on the other hand, accumulated in plants of *P19-GUS* line 1 by an average of 21.2-fold more than in those of *P19-GUS* line 3 (Figure 4a). At the DNA level, *P19-GUS* line 1 displayed a simpler profile than *P19-GUS* line 3, which showed a multiple loci integration pattern of the *P19* and *GUS* genes (data not shown).

To further investigate the contrasting results observed with the *P19-GUS* transgenic line 3 (expressing high *P19*, but low *GUS* levels), we checked for the presence of any possible mutation in the *P19* gene as well as for the methylation status of the coding region of *P19* and *GUS* and of the Ubi promoter driving the expression of both genes. The *P19* gene, derived from cDNA synthesized from RNA of each of the three plants of *P19-GUS* line 3, did not have any mutations (data not shown). The methylation of the *GUS* gene (driven by the Ubi promoter) and the Ubi promoter (driving *P19* or *GUS*) was assessed by Southern blot hybridization, using genomic DNA from two representative plants of *P19-GUS* line 3 digested with methylation-sensitive *Hpa*II (H), and methylation-insensitive *Msp*I (M), restriction endonucleases, and probes for the *GUS* coding region and the Ubi promoter, respectively. As shown in Figure 4b, the *P19-GUS* line 3 plants exhibited a high level of methylation of the *GUS* gene and the Ubi promoter, compared to their unmethylation status in the *P19-GUS* line 1 plants (expressing high *GUS* and low *P19* levels); the majority of the hybridizing *Hpa*II fragments in line 3 were of higher molecular weight. Methylation of the *P19* gene was also revealed in the *P19-GUS* line 3 plants when Southern blot hybridization was performed using the *P19* gene as a probe (data not shown).

## Discussion

### Reproducible Transient Expression Systems for the Rapid Screening for Functional RNA Silencing Suppressors in Sugarcane

Transient gene expression is influenced by several factors including species and physiological status of the explant [52], transformation parameters [53], [54], timing of gene integration [55], cell death and loss of DNA [56], [57], and gene structure [58]. PTGS also plays an important role in the post-introduction gene expression decline, so-called transient expression [39], [59].

In the present study, we have established two simple and reproducible transient expression systems for screening for functional viral RNA silencing suppressors in sugarcane. The first transient system is based on the co-expression of the suppressor and the target gene *in vivo* in young leaf segments via particle bombardment. It provides an easy and rapid evaluation of the suppressor activity due to the simplicity of the transformation method and the type of target tissue that offers a large and homogeneous surface for detection of the *EYFP* reporter gene. The use of image analysis was important to asses the *EYFP* expression level over time by determining indexes such as foci count, *EYFP* expression (gray values×pixel number) and total expression levels (combination of gray values×pixel number in green and red channels) (ImageJ software), and to correlate them with the levels of transgene protection by viral suppressors. This assay system is non-destructive and has been previously shown to be useful in sugarcane for studying efficiencies of terminators [58], and in lima bean for analyzing the effect of RNA silencing suppressors [39], [59] and the activity of a soybean promoter [60].

Our establishment of a second transient expression system, based on sugarcane protoplasts transfected via PEG and using the *GUS* reporter gene, has allowed us to study the efficiency of RNA silencing suppressors in a single cell and over time. Although the isolation of sugarcane protoplasts was first achieved in the 1970s [61], and several scientists have reported their subsequent use in plant regeneration [62], [63] and stable transformation by electroporation or by PEG [64]–[66], this is considered to be the first established sugarcane protoplast-based RNA silencing assay. Quantitative analysis of *GUS* made it possible to correlate the suppressor activity with the efficiency of silencing suppression at the cellular level over time. The protoplast cells are considered to be more homogeneous than the cells within a plant and allow the collection of consistent data for a detailed time course analysis of the suppressor activity. The high yield of viable isolated protoplasts as well as the improved transfection efficiency played key roles in the establishment of the sugarcane protoplast system for transient monitoring of the efficiency of silencing suppressors. The 30% transfection efficiency of sugarcane protoplasts achieved in this study is very acceptable as compared to the 50–70% protoplast transfection efficiency in rice [67].

The protoplast-based RNA silencing assay offers unique advantages to study the mechanisms of RNA silencing in protoplast cells. However, some of the differing results between protoplasts and *in planta* studies, which are caused by protoplasts as single cells or by the cells within a plant body, may have biological differences that affect the RNA silencing pathway [36]. Therefore, it is necessary to have a combination of single cell and *in planta* studies to generate information on the kinetic features of the RNA silencing suppressors.

### Enhancement of Transgene Expression in Sugarcane by the use of the P19 Viral RNA Silencing Suppressor

In the present study, five viral RNA silencing suppressors, *P19*, *P19/R43W*, *γb*, *P1/HC-Pro* and *SCBV OrfI*, were co-expressed transiently with the *EYFP* or *GUS* reporter gene in sugarcane leaf segment tissues and protoplasts in order to investigate their activity as suppressors of post-transcriptional transgene silencing. Although these suppressors have been well studied previously, the primary transient expression assay system used relied on *Agrobacterium*-infiltration of *GFP* into model plant systems like *N. benthamiana* leaves [16], as verified in the present study (Figure S1). However, several monocot species, including sugarcane, are not amenable to *Agrobacterium*-infiltration, thus requiring alternative approaches for transient studies. We demonstrated that the viral RNA silencing suppressors operate in sugarcane leaf segments and protoplasts by co-bombardment or co-transfection with the target reporter genes, *EYFP* and *GUS*. Our findings indicated that P19, P19/R43W and γb suppressors contributed to increased and extended *EYFP* or *GUS* expression, and this increase was highly dependent on the system used. In young leaf segments, P19 and γb significantly enhanced *EYFP* expression and extended its peak to over 240 h post-bombardment (Figure 2). In protoplasts, P19, P19/R43W and γb resulted in a significant increase in GUS activity until 24 h post-transfection (Figure 3). The fact that P19 and γb worked well in our systems indicate that siRNA sequestration is probably more effective for suppression than inhibiting the RISC complex, as is the case with P1/HC-Pro that did not have any effect [1], [20], [68]. The ability of P19, P19/R43W and γb to enhance transgene expression in transient assays has been well documented in *N. tabacum* and *N. benthamiana* [20], [36], [46], but using *Agrobacterium*-mediated delivery of these suppressors.

The effect of a viral suppressor on the expression of the co-introduced *EYFP* or *GUS* was found to be dependent on the dose of the co-delivered suppressor in our sugarcane transient expression system. Co-expression of *EYFP* with increasing doses of the *P19* suppressor was noted to enhance *EYFP* expression and prolong the *EYFP* expression peak by at least 120 h in young leaf segments (Figure 1). Similarly, an increase in GUS activity was achieved in protoplasts by increasing the amount of co-transfected *P19* (Table 1). These results confirm a previous report where the suppression activity of P19 was dosage dependent in *N. benthamiana* [69].

The sugarcane transgenic independent lines co-expressing the *GUS* reporter and the suppressor *P19* generated in the present study showed a significant (*p*<0.05) enhancement in their GUS activity levels by 1.9-fold to 3.5-fold more than those expressing *GUS* alone (Table 2). The *P19-GUS* transgenic plants were noted to develop normally, with no detrimental phenotypic effects, indicating that P19 was tolerated within the stably transformed sugarcane plants. This is an improvement to the transgenic expression of *P19* in *A*. *thaliana*, *N*. *tabacum* and *N*. *benthamiana* that often yielded plants with deformed phenotypes [31], [46], [51]. While we can not rule out other possibilities, the normal development and growth in the *P19-GUS* transgenic lines is consistent with the low expression levels of *P19* (Figure 4a).

The majority of the *P19-GUS* transgenic sugarcane plants exhibited high *GUS* levels with a low detectable *P19* expression level (Figure 4a) to avoid plant toxicity, possibly due to the use of the strong constitutive Ubi promoter. The methylation status of the Ubi promoter as well as that of *GUS* and *P19* genes in one silenced line certainly adds to the understanding of this phenomenon. Among the seven *P19-GUS* transgenic lines characterized in this study, line 3 (silenced line), represented by three plants, exhibited low *GUS* transcript level and activity (Figure 4a). We envision at least two possible reasons for the lower *GUS* expression levels. One is that P19 is defective or less efficient. Alternatively, GUS expression is silenced at the step(s) that can not be overcome by P19. We cloned the *P19* cDNAs derived from RNA of each of the three plants of line 3. Among 5 of the cDNA clones, we did not find any mutations, indicating that the *P19* transcript was not altered. Conversely, the *P19-GUS* line 3 exhibited a high level of methylation in both the *GUS* gene and the Ubi promoter, compared to their unmethylation status in the non-silenced *P19-GUS* line 1 expressing high *GUS* levels (Figure 4b).

Two types of transgene-induced gene silencing are known to exist in plants [70], [71]. One type acts at the transcriptional level (TGS), through repression of transcription, where the transgene possesses sequence homology to the promoter of the silenced gene, and the other type works at the PTGS level, through mRNA degradation, requiring homology in the transcript. The TGS is often associated with increased DNA methylation, while PTGS involves sequence-specific RNA degradation, although methylation in either coding or transcribed regions of silenced transgenes has been detected in many cases of PTGS [72]–[74]. The methylation of the coding region of the *GUS* gene in the *P19-GUS* line 3 (Figure 4b) is more likely to be associated with PTGS, while the Ubi promoter methylation (Figure 4b) is related to TGS. The *P19* suppressor is probably not functional due to its silencing at the PTGS level, while *GUS* is silenced at the TGS and PTGS levels. This data is consistent with the methylation of the zein gene and the phaseolin promoter in silenced transgenic soybean lines [75].

### Conclusion

Two transient transgene expression assays based on young leaf segments and protoplasts, and confirmed by stable transgene expression, were successfully established in the present study to provide a rapid and reproducible versatile system to screen for functional RNA silencing suppressors in sugarcane and other plant species. This system is the first to be developed in sugarcane, and it combines *in vivo*, single cell and *in planta* studies to generate more information on the kinetics of the suppressor activities.

The use of RNA silencing suppressors, specifically the TBSV-encoded P19 suppressor, proved to be an efficient strategy in allowing for high levels of foreign protein production, whether by transient or stable transgene expression, to counteract the deleterious effects of RNA silencing in sugarcane. This approach provides a suitable platform for the cost-effective production of high-value recombinant proteins for the exploitation of a variety of biotechnologically attractive plant species, such as sugarcane and other high biomass producers, as biofactories. Researchers have previously used the *Artichoke mottled crinkle virus*-encoded P19 suppressor in *Agrobacterium* infiltration transient gene expression systems to produce high yields of biopharmaceuticals, namely a human antibody against the tumour-associated antigen tenascin-C [76] and the HIV-1 Nef protein [77] in *N. tabacum* and *N. benthamiana*, respectively.

## Materials and Methods

### DNA Constructs

The four RNA silencing suppressors, the *Tobacco etch virus*-encoded P1/HC-Pro, the *Barley stripe mosaic virus*-encoded encoded γb, and the *Tomato bushy stunt virus*-encoded P19 and its mutant P19/R43W were kindly provided by colleagues (Legend, Figure S1). The putative suppressor OrfI was cloned from *Sugarcane bacilliform virus* (SCBV) isolates in our laboratory at Texas A&M AgriLife Research (Weslaco, Texas). All the suppressor genes were cloned into the vector pAHC20 [78] with no *BAR* gene, named pUbi-ALS, under the control of the maize *ubiquitin 1* (Ubi) promoter and the *Agrobacterium tumefaciens* nopaline synthase terminator (Tnos) (Figure S3). The *P1/HC-Pro* fragment was obtained from pGD-*TEV* [26] by digestion with *Xho*Ι and *Bam*HI restriction endonucleases; it included 133 bp of 5′UTR, *P1/HC-Pro* (2289 bp) and partial *P3* (248 bp). The *γb* fragment (527 bp) was released from pGD-*γb* [26] with *Xho*Ι and *Pst*I. The *P19* and *P19/R43W* fragments (617 bp each) were excised with *Nco*Ι and *Sal*Ι from pUC19-wt *TBSV P19* and pUC19-*P19/R43W* [68], respectively. P19/R43W contains one point mutation at nucleotide 127 where C has been replaced with T [46], [68]. The *OrfI* fragment (593 bp) of SCBV was amplified by PCR using the primers SCBVOrfI*Xho*ΙHis-F (5′-ccg*ctcgaga*tgcatcaccatcaccatcacaaaaccgaatctgagtgg-3′) and SCBVOrfI*Bam*HΙ-R (5′-cg*ggatcc*ttagctgatacgtttcaccatgtg-3′) and cloned into pGEM-T Easy (Promega, Madison, WI) to yield the pGEM/*SCBVOrfI* plasmid. The pUbi-ALS plasmid was linearized by *Sal*Ι to generate the pUbi:Tnos cassette, which consists of pUC8 with the Ubi promoter and Tnos terminator. Subsequently, the five suppressor fragments were blunt ended using DNA Polymerase Ι, Large (Klenow) Fragment (New England BioLabs Inc., MA), and cloned into the linearized pUbi:Tnos cassette. Identity of all assembled constructs was verified by sequencing. The pUbi:*GUS* (pAHC27) [78] and pUbi:*EYFP*:Tnos [58] constructs (Figure S3) were used for the expression of the *GUS* and *EYFP* reporter genes, respectively.

### Target Tissue, DNA Particle Bombardment and Transgenic Plants

Stalk tops of field-grown sugarcane (*Saccharum* spp. hybrid, commercial variety CP72-1210) were collected during the growing season at the Texas A&M AgriLife Research Annex Farm (Weslaco, Texas). No specific permits were required for the described field study and the location, and the location is not privately owned or protected in any way. The field study did not involve endangered or protected species. Young leaf segments were prepared from the sugarcane stalk tops for transient gene expression assays as described by Beyene *et al* [58]. Briefly, leaf blades and sheaths were removed down to leaf 1 (the top visible dewlap leaf), and the upper 20–30 cm portion of shoot (leaf roll stalk) was surface sterilized in 70% (v/v) ethanol for about 20 min. The two outermost leaf sheathes were discarded, and leaves at position -3 were unfolded, their midribs removed and their blade cut into about 2.5 cm^2^ leaf segments. For transient gene expression, segments were pre-cultured adaxial side down onto MS medium [79] with 2,4-dichlorophenoxyacetic acid (2,4-D) at 0.6 mg/L (MS0.6 medium) [80] and casein hydrolysate (500 mg/L) for 3 days in the dark at 28°C before DNA particle bombardment.

For stable transgene expression, sugarcane leaf roll discs were obtained from the stalk tops as described for leaf segments, and used as explants for DNA particle bombardment. Immature leaf rolls close to the apical meristem were sliced transversely into 1 mm thick sections and cultured on MS0.6 medium for 10–28 days. Leaf roll discs were preconditioned on MS0.6 osmoticum (MS0.6 with 36.44 g/L of D-mannitol and 36.44 g/L of D-sorbitol) for 4 h prior to and after DNA particle bombardment. Bombarded leaf roll discs were maintained on MS0.6 medium for 7 days in the dark at 28°C for recovery. They were later broken into small pieces and incubated in the dark at 28°C on callus induction medium, MS0.6 with Bialaphos (4 mg/L) selection, for a total of 4 weeks, with sub-culturing every two weeks. For shoot regeneration, calli from leaf roll discs were grown on MS supplemented with kinetin (2 mg/L), naphthalene acetic acid (2 mg/L) and Bialaphos (4 mg/L) for 6–8 weeks under a light (16 h)/dark (8 h) photoperiod. Green shoots of approximately 2 cm in height were transferred into MS rooting medium containing indole-3-butyric acid (4 mg/L) and Bialaphos (4 mg/L). Rooted plantlets were transferred to potting soil (Metromix, Scotts, Hope, AR) in pots and maintained in the greenhouse.

DNA coating for particle gun bombardment was performed according to Beyene *et al* [58]. Briefly, tungsten particles (M17, 1.1 micron; Bio-Rad Laboratories, Hercules, CA) were sterilized in absolute ethanol and resuspended in nuclease-free water to a final concentration of 60 ng/µL following the manufacturer’s instructions. Plasmid DNA was precipitated onto tungsten particles at a concentration of 2.0 µg (*GUS*; for stable expression) or 0.5 µg (*EYFP*; for transient expression) DNA per mg of tungsten using calcium chloride (CaCl_2_) (2.5 M) and spermidine (0.1 M). The molecular ratio of the *GUS* or *EYFP* plasmid to the suppressor plasmid was 1∶2. The DNA-coated tungsten particles were resuspended in 40 µL of absolute ethanol, and 4 µL of this suspension was used per bombardment of the target tissue (leaf segment or leaf roll). For DNA particle discharging, a modified particle inflow gun [81] with helium gas (110 psi) was used, as described previously [52].

### Protoplast Isolation and Transfection

Protoplasts were isolated from suspension cell cultures of callus originating from sugarcane immature leaf roll discs using the modified methods of Chen *et al* [64] and Yoo *et al* [33]. Briefly, suspension cell cultures (100 mL) were maintained on a rotary shaker (250 mL flasks; 100 rpm) by weekly subculturing (1∶5 dilution) in MS liquid medium with 2,4-D (3.0 mg/L). The freshly harvested suspension cells (subcultured for 2–3 days) were incubated overnight at room temperature in enzyme solution [20 mM MES (pH 5.7), 2.0% (w/v) Cellulysin® cellulase (EMD Biosciences, San Diego, CA), 0.1% (w/v) pectolyase Y-23 (Duchefa Biochemie, St. Louis, MO), 0.4 M D-mannitol, 20 mM potassium chloride (KCl), 10 mM CaCl_2_ and 0.1% (w/v) bovine serum albumin]. Protoplasts were washed twice in W5 solution (2 mM MES, 154 mM sodium chloride, 125 mM CaCl_2_ and 5 mM KCl), pelleted at 100×*g* for 2 min, and suspended in 4 mM MES-KOH (pH 5.7), 0.4 M D-mannitol and 15 mM magnesium chloride at a final concentration of 1×10^6^ protoplasts per mL.

Transfection of protoplasts with plasmid DNA was performed according to Yoo *et al* [33]. Briefly, protoplasts (1×10^5^; 100 µL) were transferred into a 2-mL microcentrifuge tube (round-bottom) and mixed gently with plasmid DNA (5 µg of *GUS* reporter plasmid and 10 µg of suppressor plasmid in 10 µL volume). Equivalent volumes of sterile water (mock-transfection) and empty vector (pUbi-Tnos) were used as controls for transfection. Protoplasts were mixed gently with a PEG-calcium solution [40% polyethylene glycol-4000 (PEG), 0.2 M D-mannitol and 100 mM CaCl_2_] (110 µL) and incubated for 10 min at room temperature. Transfection was terminated by the dilution of the mixture in W5 solution (440 µL). Transfected protoplasts were collected by centrifugation for 2 min at 100×*g* and suspended in W5 solution (250 µL). *GUS* expression was analyzed after incubation of the protoplasts in the dark for 24 h at room temperature. The number of protoplasts expressing *EYFP* was determined manually using a SZX7 fluorescence stereomicroscope with a DP71 cooled CCD camera (Olympus, Center Valley, PA) and a YFP filter (85.5x magnification).

### Southern Blot, Northern Blot and Quantitative RT-PCR Analyses

Genomic DNA and total RNA were isolated from liquid nitrogen-ground leaf tissues (0.5–1 g fresh weight) collected from young leaves of 3–4 month-old sugarcane transgenic plants according to Tai and Tanksley [82] and Damaj *et al* [83], respectively.

Genomic DNA (10 µg per lane) was digested overnight with either *Hind*III, *Msp*I or *Hpa*II, electrophoresed on 0.8% (w/v) agarose gels and transferred to nylon membranes (Amersham Hybond-XL, GE Healthcare Bio-Sciences Corp., Piscataway, NJ) in an alkaline solution (0.4 M sodium hydroxide) [84]. Total RNA (15 µg per lane) was fractionated on 1.6% formaldehyde agarose denaturing gels in HEPES buffer and blotted onto nylon membranes (Amersham Hybond-XL) in 10x SSC [85].

Pre-hybridization, hybridization, washing and detection of DNA and RNA gel blots were performed as described by Sambrook *et al* [86] and Mangwende *et al* [85], using Church’s buffer. The *GUS*-specific probe was obtained from pUbi:*GUS* (Figure S3) by *Bbs*I and *Sac*I digestion, and all of the five RNA silencing suppressor probes were prepared from their respective constructs (Figure S3) after digestion with *Pst*I. For methylation analysis, DNA probes were obtained by further digesting the *GUS*-specific probe with *Msp*I into 8 fragments, and by releasing the Ubi promoter from pUbi:*EYFP*:Tnos (Figure S3) with *Hind*III and *Nco*I. Probes were labeled with [α-^32^P] dCTP using the Random Primers DNA Labeling kit (Invitrogen, Carlsbad, CA) [85].

### Analysis of β-glucuronidase Activity

Quantitative β-glucuronidase (GUS) assays were performed on transfected protoplasts and leaf tissue of transgenic plants using 4-methylumbelliferyl-β-D-glucuronide (MUG) as a fluorescent substrate [87].

Transfected protoplasts were harvested by centrifugation at 100×*g* for 2 min, and stored at -80°C until analysis. Frozen protoplasts were ruptured in GUS extraction buffer (50 mM sodium phosphate buffer pH 7.0, 10 mM 2-mercaptoethanol, 10 mM EDTA pH 8.0, and 0.1% [v/v] Triton X-100) (100 µL) by vortexing for 2 s, and incubated on ice for 5 min. Total soluble protein extracts were collected by centrifugation at 1000×*g* for 2 min at 4°C.

Leaf tissue (500 mg) of transgenic plants (3–4 month-old), ground into powder in liquid nitrogen, was suspended in GUS extraction buffer (750 µL) by brief vortexing and incubated on ice for 1 h. Total soluble protein extracts were collected by centrifugation at 12,000×*g* for 10 min at 4°C.

Fluorometric GUS assay was carried out on total soluble protein extracts from protoplasts (25 µL) and leaf samples (10 µL of extract and 15 µL of GUS extraction buffer) in 4 mM MUG assay buffer (25 µL) by incubation for 60 min at 37°C. The reaction was stopped by the addition of 0.2 M sodium carbonate (950 µL). Fluorescence was measured at 455 nm (emission) and 365 nm (excitation) using a VersaFluor™ Fluorometer (Bio-Rad Laboratories). Protein concentrations were determined by the Lowry assay method using the DC protein assay kit (Bio-Rad Laboratories). Protein extracts from protoplasts transfected with empty vector and from leaves of non-transgenic plants were used as a negative control.

### EYFP Imaging and Analysis

Images (4,080×3,072 pixels) of sugarcane young leaf segments expressing *EYFP* were collected every 6 h post-bombardment for at least 240 h by a SZX7 fluorescence stereomicroscope with a DP71 digital camera (Olympus) fitted with YFPHQ filters (excitation of 490–500 nm and emission of 515–560 nm) under 15x magnification. *EYFP* expression was quantified using the ImageJ version 1.42u software according to the revised method of Chiera *et al* [59], [60] and as described by Beyene *et al* [58]. Briefly, a series of images taken over time from each sample were saved in separate folders and imported into Adobe® ImageReady™ as frames. The sequence of images was then resized to 800×600 pixels and exported as “mov” files. For image analysis, “mov” files were imported into the ImageJ software, and a representative 400×300 pixel area was selected and cropped. This 400×300 pixel area was used as the original image for calculating the *EYFP* foci count and *EYFP* expression level. Subsequently, all image series were separated into red, green and blue channels and their background was corrected. For further analysis, only the green channel was used, since the contribution of the red channel was found to be lower than 5% of total *EYFP* expression. Plugins for quantification of *EYFP* expression and foci count were kindly provided by J. Chiera and C. Hernandez-Garcia (Department of Horticulture and Crop Sciences, The Ohio State University, Columbus, Ohio). *EYFP* expression was calculated by multiplying the mean grayscale value per pixel by the number of *EYFP* expressing pixels with the resulting *EYFP* expression values being unitless. The number of *EYFP* expressing foci was determined by counting the number of spots bigger than ten pixels.

### Statistical Analysis

Data were collected from 2–3 independent experiments, with 6–10 replicates per experiment, and subjected to an analysis of variance (ANOVA) using the General Linear Model procedure of the Statistical Analysis System 8.1 (SAS Institute Inc., Cary, NC). Mean separation was performed using the Student-Newman-Keuls (SNK) test.

## Supporting Information

Figure S1 **Comparison of suppressors in** ***Nicotiana benthamiana.*** The plasmids carrying the silencing suppressors P1/HC-Pro (HcPro) and γb are pGD binary vectors specifically generated to be used with *Agrobacterium*, and modified from the binary vector pCAMBIA 1303, with a multiple cloning site downstream of a *Cauliflower mosaic virus* 35S promoter, and upstream of an *Agrobacterium* nopaline synthase poly(A) signal [88], [89]. P19 is expressed from the binary vector pCass4N, a derivative of a pBin19 binary vector [46]. The plasmid carrying the Green Fluorescent Protein (*GFP*) gene is 35S-*GFP* (provided by David C. Baulcombe, University of Cambridge, Cambridge, UK) [90]. All of the silencing suppressors were infiltrated into *Nicotiana benthamiana* using the *Agrobacterium* strain EHA [91], which exhibited a less virulent host response than others (data not shown). Three week-old *N. benthamiana* plants were infiltrated with 35S*-GFP* and silencing suppressors at an optical density of 0.8, mixed as indicated. The plants were then photographed at different days post-infiltration (dpi) under a 488 nm wavelength UV light with a 4 s exposure and no flash, to monitor the levels of the fluorescent GFP expressed. The plants in the first column, labeled EHA, are those infiltrated with untransformed *Agrobacterium*, as a negative control. When necessary, supplementation with *Agrobacterium* EHA was done to ensure that each leaf was inoculated with 0.5 mL of bacterial culture. For each suppressor, the expression was verified by immuno-blotting (data not shown).
(TIF)

Figure S2 **Suppression of silencing in onion epidermal cells.** (a) Example of the expression of the gene encoding the Enhanced Yellow Fluorescent Protein (EYFP) in a single onion cell at two days post-bombardment. At 1–2 h before transformation, onion epidermal peels were prepared under sterile conditions using pointed forceps and placed adaxial side up onto Murashige and Skoog basal salt mixture (MS) media [79] with 0.2 M D-mannitol and 0.2 M D-sorbitol (MS osmoticum). Two explants were used per plate, and each plate was replicated 4–5 times. Genes encoding the suppressors were under control of the maize *ubiquitin 1* promoter, as described in Materials and Methods. Plasmid DNA of the appropriate construct was introduced into onion cells using a PDS-1000/He particle delivery system. Bombardment was performed at 9 cm from targets using gold particles (1.0 micron; Bio-Rad Laboratories) coated with plasmids expressing *EYFP* or viral suppressors under 27 inch Hg and 1100 psi helium pressure. Plasmid DNA was precipitated onto the gold particles using calcium chloride (2.5 M) and spermidine (0.1 M). For co-introduction of two and three different plasmids, 4.5 µg and 3.0 µg of each plasmid was used, respectively. The bombarded epidermal peels were incubated on MS osmoticum for 48–72 h at 25°C in the dark. Fluorescence was monitored using a fluorescence binocular microscope Olympus SZX10 with an excitation wavelength of 490 nm. (b) Comparison of different suppressors. Similarly sized onion peel sections were bombarded as described for (a), and the number of fluorescent cells was counted.
(DOC)

Figure S3 **Map of suppressor and reporter gene constructs for stable sugarcane transformation.** For genetic construct assembly, refer to Materials and Methods. P1/HC-Pro is derived from *Tobacco etch virus*, γb from *Barley stripe mosaic virus*, P19 and P19/R43W, a mutant of P19, from *Tomato bushy stunt virus*, and SCBV OrfΙ from *Sugarcane bacilliform virus;* GUS: β-glucuronidase; EYFP: Enhanced Yellow Fluorescent Protein; pUbi: Maize *ubiquitin 1* promoter; Tnos: *Agrobacterium tumefaciens* nopaline synthase terminator. Boxes are not drawn to scale.
(TIF)

Figure S4 **Multicolor fluorescence imaging of cells in sugarcane young leaf segments expressing the** ***EYFP*** **reporter gene, under low optical magification.** Images of *EYFP* expression were collected with a SteReo Lumar.V12 fluorescence stereomicroscope and an AxioCam ICc3 digital camera (15x magnification) (Carl Zeiss) from young leaf segments at 48 h after bombardment with *EYFP* (pUbi:*EYFP*:Tnos; Figure S3) (0.5 µg per shot) (*bar* = 0.5 mm). Vector with no *EYFP* (pUbi:Tnos; Figure S3) and water were used as negative controls. Images were taken under bright light as well as with filters for rhodamine (filter model FS20) (excitation: 546/12 nm, emission: 575–640 nm), eGFP (filter model FS38) (excitation: 470/40 nm, emission: 525/50 nm) and YFP (filter model FS46 HE) (excitation: 500/25 nm, emission: 535/30 nm). eGFP and YFP images were taken under 700 ms exposure, and rhodamine images were taken under autoexposure.
(TIF)

Figure S5 **Multicolor fluorescence imaging of cells in sugarcane young leaf segments expressing the** ***EYFP*** **reporter gene, under high optical magnification.** Images of *EYFP* expression were collected with a SteReo Lumar.V12 fluorescence stereomicroscope and an AxioCam ICc3 digital camera (150x magnification) (Carl Zeiss) from young leaf segments at 48 h after bombardment with *EYFP* (pUbi:*EYFP*:Tnos; Figure S3) (0.5 µg per shot) (*bar* = 0.05 mm). Vector with no *EYFP* (pUbi:Tnos; Figure S3) and water were used as negative controls. Images were taken under bright light as well as with filters for rhodamine (filter model FS20) (excitation: 546/12 nm, emission: 575–640 nm), eGFP (filter model FS38) (excitation: 470/40 nm, emission: 525/50 nm) and YFP (filter model FS46 HE) (excitation: 500/25 nm, emission: 535/30 nm). Overlaid images were generated from merged images of bright light and YFP filter. eGFP and YFP images were taken under 700 ms exposure, and rhodamine images were taken under autoexposure.
(TIF)

Figure S6 **Quantitative assessment of transient expression of the** ***EYFP*** **reporter gene in sugarcane young leaf segments.** *EYFP* expression as measured by foci count (a) and expression level (b) was monitored in 3 day-pre-cultured young leaf segments at 48 h after bombardment with *EYFP* (pUbi:*EYFP*:Tnos; Figure S3) (0.5 µg per shot). Vector with no *EYFP* (pUbi:Tnos; Figure S3) and water were used as negative controls. Values represent means with standard error from three independent experiments and 10 replicates per experiment. Means with the same letter are not significantly different (*p*>0.05). Quantitation of *EYFP* foci counts and expression levels is provided in Materials and Methods.
(TIF)

Figure S7 **Transient expression of the** ***EYFP*** **reporter gene in sugarcane protoplasts.** (a) A seven day-old leaf roll disc growing on MS shoot regeneration medium (9.5x magnification, *bar* = 1.0 mm); (b) Callus regenerated from leaf roll disc after subculture on MS medium for 4–6 weeks and grown in MS liquid medium to obtain suspension cells (12x magnification, *bar* = 1.0 mm); (c) Protoplasts isolated from suspension cells under bright light (400x, *bar* = 20 µm); (d) and (e) Protoplasts transfected with the pUbi:*EYFP*:Tnos vector expressing *EYFP* under bright light (100x magnification) and EYFP filter (100x magnification), respectively (*bar* = 100 µm); (f) Overlaid image of (d) and (e) showing transfection efficiency; a transfected protoplast is indicated by an arrow (*bar* = 100 µm). Microphotographs of (a) and (b) were collected using an Olympus SZX7 fluorescence microscope with a DP71 camera. Microphotographs of (c), (d), (e) and (f) were obtained with an Olympus BX51 fluorescence microscope with a DP72 camera.
(TIF)

Figure S8 **Effect of viral RNA silencing suppressors on transient expression of the** ***EYFP*** **reporter gene in sugarcane young leaf segments.** Images of *EYFP* expression were collected with a SZX7 fluorescence stereomicroscope and a DP71 digital camera (15x magnification) (Olympus) from the same young leaf segments at 24–240 h after co-bombardment with *EYFP* (pUbi:*EYFP*:Tnos; Figure S3) (0.25 µg) and each of the RNA silencing suppressors (driven by the Ubi promoter, Figure S3), *HC-Pro*, *γb*, *P19*, *P19/R43W* and *SCBV OrfI* (0.5 µg) (*bar* = 0.5 mm). Vector with no suppressor (pUbi:Tnos; Figure S3) was used as a negative control.
(TIF)
