# Supplementary figures and images for: Enhanced Transgene Expression in Sugarcane by Co-Expression of Virus-Encoded RNA Silencing Suppressors
Source: PLoS One. 2013 Jun 14;8(6):e66046. doi: 10.1371/journal.pone.0066046 (PMC3682945; doi:10.1371/journal.pone.0066046)

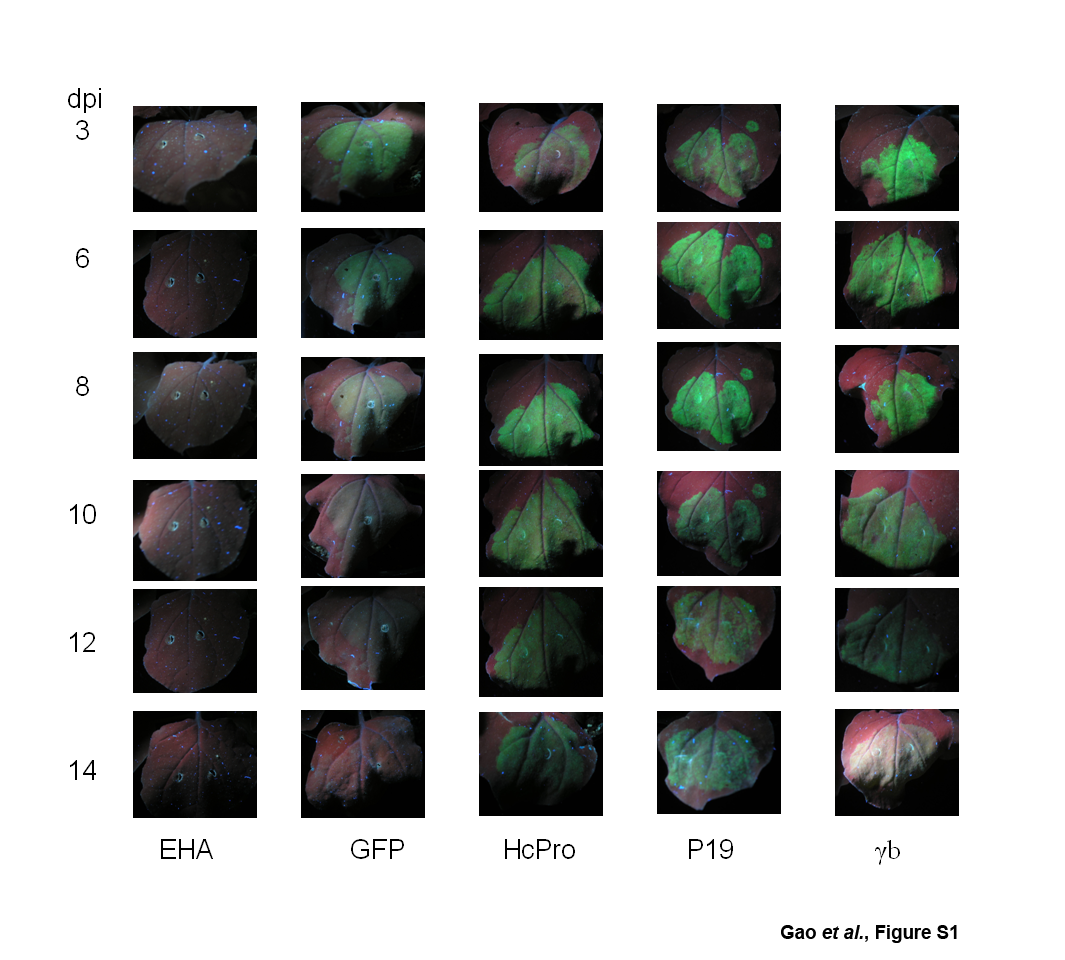

Supplement: Figure S1 — Comparison of suppressors in Nicotiana benthamiana. The plasmids carrying the silencing suppressors P1/HC-Pro (HcPro) and γb are pGD binary vectors specifically generated to be used with Agrobacterium, and modified from the binary vector pCAMBIA 1303, with a multiple cloning site downstream of a Cauliflower mosaic virus 35S promoter, and upstream of an Agrobacterium nopaline synthase poly(A) signal [88], [89]. P19 is expressed from the binary vector pCass4N, a derivative of a pBin19 binary vector [46]. The plasmid carrying the Green Fluorescent Protein (GFP) gene is 35S-GFP (provided by David C. Baulcombe, University of Cambridge, Cambridge, UK) [90]. All of the silencing suppressors were infiltrated into Nicotiana benthamiana using the Agrobacterium strain EHA [91], which exhibited a less virulent host response than others (data not shown). Three week-old N. benthamiana plants were infiltrated with 35S-GFP and silencing suppressors at an optical density of 0.8, mixed as indicated. The plants were then photographed at different days post-infiltration (dpi) under a 488 nm wavelength UV light with a 4 s exposure and no flash, to monitor the levels of the fluorescent GFP expressed. The plants in the first column, labeled EHA, are those infiltrated with untransformed Agrobacterium, as a negative control. When necessary, supplementation with Agrobacterium EHA was done to ensure that each leaf was inoculated with 0.5 mL of bacterial culture. For each suppressor, the expression was verified by immuno-blotting (data not shown). (TIF) [file pone.0066046.s001.tif]

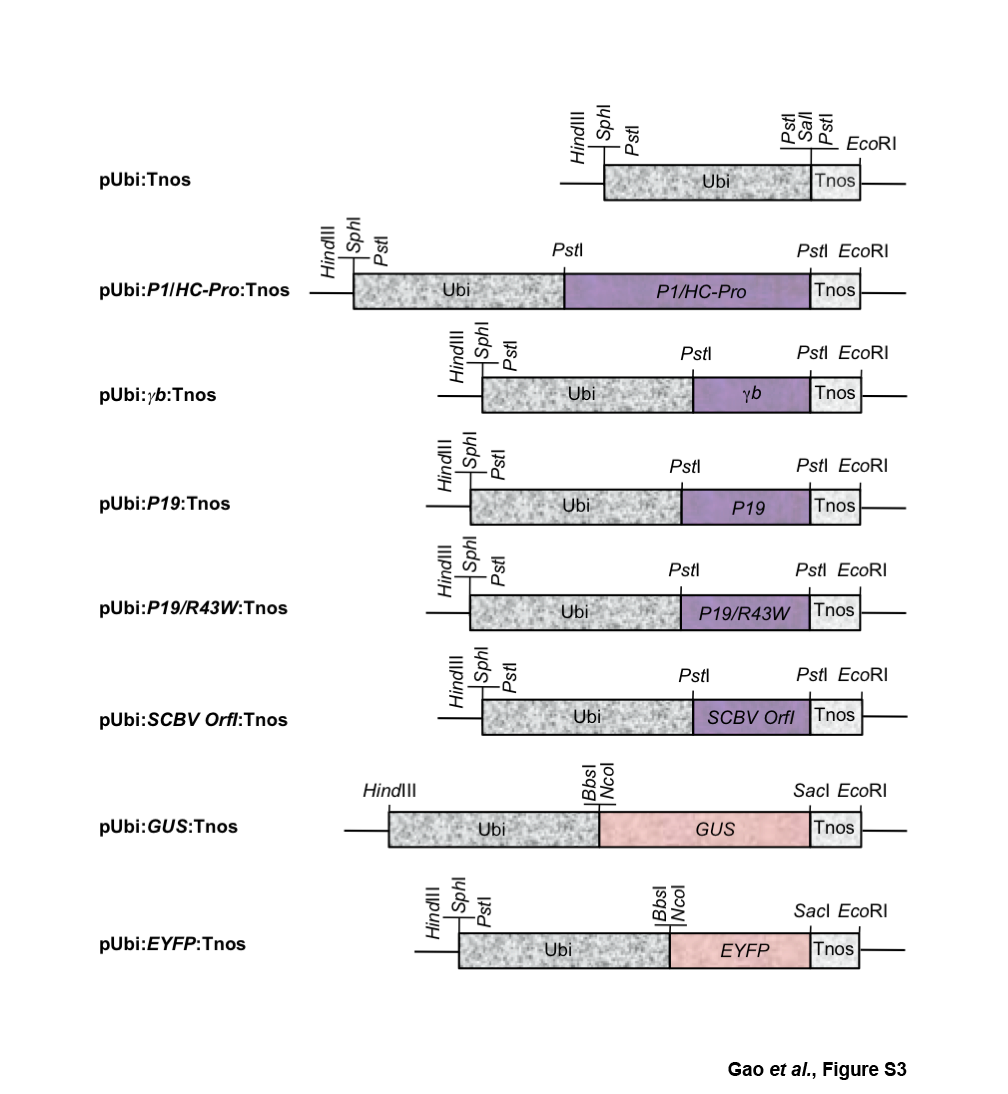

Supplement: Figure S3 — Map of suppressor and reporter gene constructs for stable sugarcane transformation. For genetic construct assembly, refer to Materials and Methods. P1/HC-Pro is derived from Tobacco etch virus, γb from Barley stripe mosaic virus, P19 and P19/R43W, a mutant of P19, from Tomato bushy stunt virus, and SCBV OrfΙ from Sugarcane bacilliform virus; GUS: β-glucuronidase; EYFP: Enhanced Yellow Fluorescent Protein; pUbi: Maize ubiquitin 1 promoter; Tnos: Agrobacterium tumefaciens nopaline synthase terminator. Boxes are not drawn to scale. (TIF) [file pone.0066046.s003.tif]

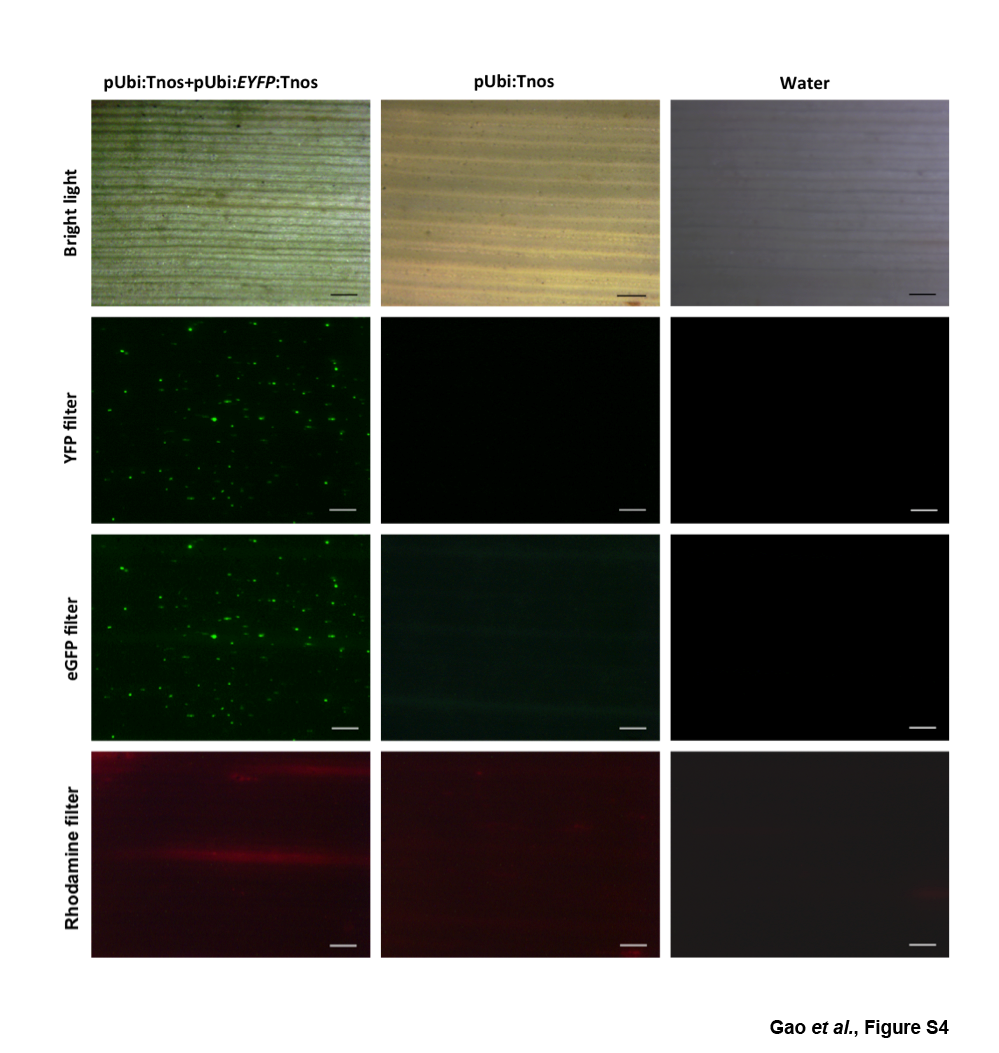

Supplement: Figure S4 — Multicolor fluorescence imaging of cells in sugarcane young leaf segments expressing the EYFP reporter gene, under low optical magification. Images of EYFP expression were collected with a SteReo Lumar.V12 fluorescence stereomicroscope and an AxioCam ICc3 digital camera (15x magnification) (Carl Zeiss) from young leaf segments at 48 h after bombardment with EYFP (pUbi:EYFP:Tnos; Figure S3) (0.5 µg per shot) (bar = 0.5 mm). Vector with no EYFP (pUbi:Tnos; Figure S3) and water were used as negative controls. Images were taken under bright light as well as with filters for rhodamine (filter model FS20) (excitation: 546/12 nm, emission: 575–640 nm), eGFP (filter model FS38) (excitation: 470/40 nm, emission: 525/50 nm) and YFP (filter model FS46 HE) (excitation: 500/25 nm, emission: 535/30 nm). eGFP and YFP images were taken under 700 ms exposure, and rhodamine images were taken under autoexposure. (TIF) [file pone.0066046.s004.tif]

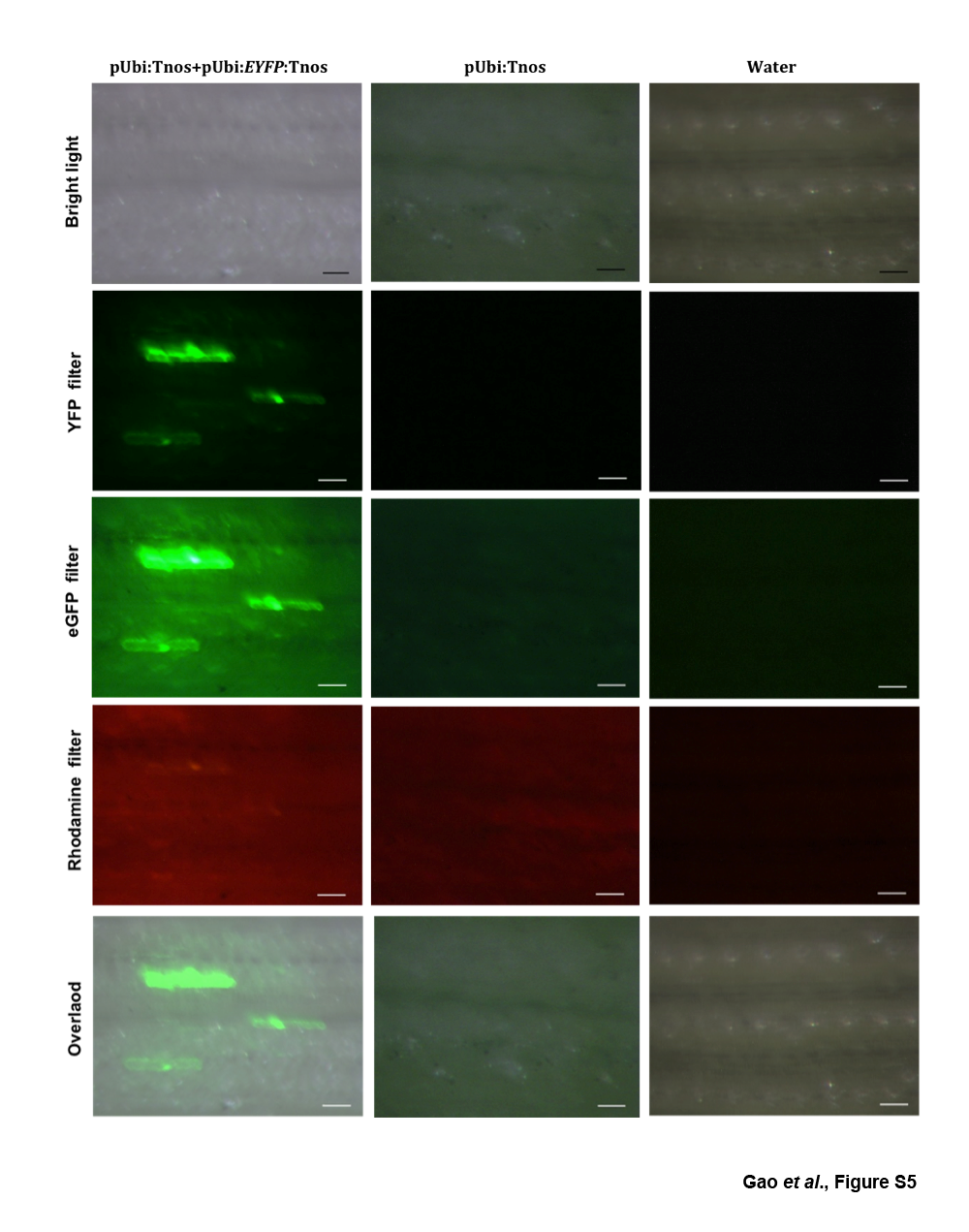

Supplement: Figure S5 — Multicolor fluorescence imaging of cells in sugarcane young leaf segments expressing the EYFP reporter gene, under high optical magnification. Images of EYFP expression were collected with a SteReo Lumar.V12 fluorescence stereomicroscope and an AxioCam ICc3 digital camera (150x magnification) (Carl Zeiss) from young leaf segments at 48 h after bombardment with EYFP (pUbi:EYFP:Tnos; Figure S3) (0.5 µg per shot) (bar = 0.05 mm). Vector with no EYFP (pUbi:Tnos; Figure S3) and water were used as negative controls. Images were taken under bright light as well as with filters for rhodamine (filter model FS20) (excitation: 546/12 nm, emission: 575–640 nm), eGFP (filter model FS38) (excitation: 470/40 nm, emission: 525/50 nm) and YFP (filter model FS46 HE) (excitation: 500/25 nm, emission: 535/30 nm). Overlaid images were generated from merged images of bright light and YFP filter. eGFP and YFP images were taken under 700 ms exposure, and rhodamine images were taken under autoexposure. (TIF) [file pone.0066046.s005.tif]

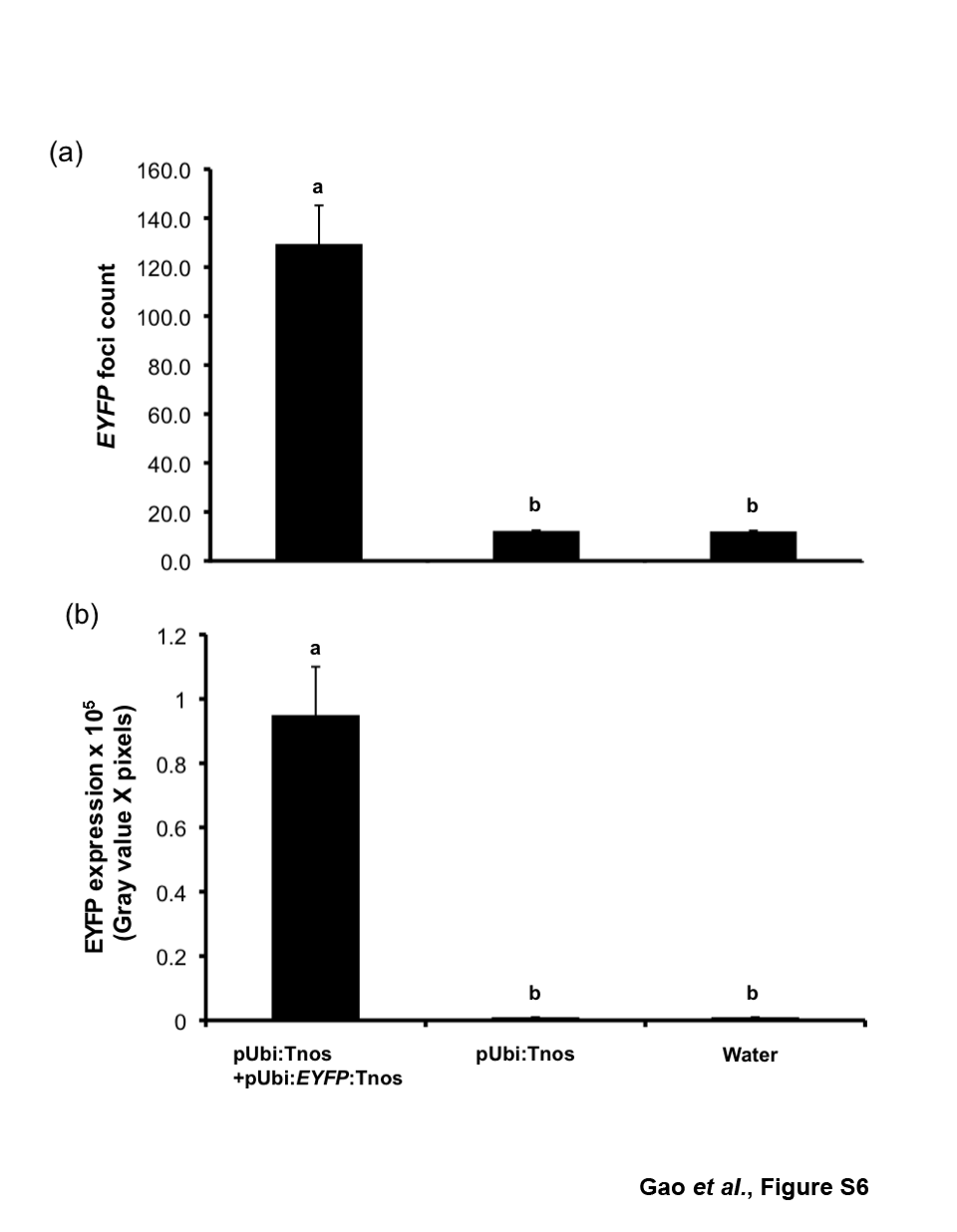

Supplement: Figure S6 — Quantitative assessment of transient expression of the EYFP reporter gene in sugarcane young leaf segments. EYFP expression as measured by foci count (a) and expression level (b) was monitored in 3 day-pre-cultured young leaf segments at 48 h after bombardment with EYFP (pUbi:EYFP:Tnos; Figure S3) (0.5 µg per shot). Vector with no EYFP (pUbi:Tnos; Figure S3) and water were used as negative controls. Values represent means with standard error from three independent experiments and 10 replicates per experiment. Means with the same letter are not significantly different (p>0.05). Quantitation of EYFP foci counts and expression levels is provided in Materials and Methods. (TIF) [file pone.0066046.s006.tif]

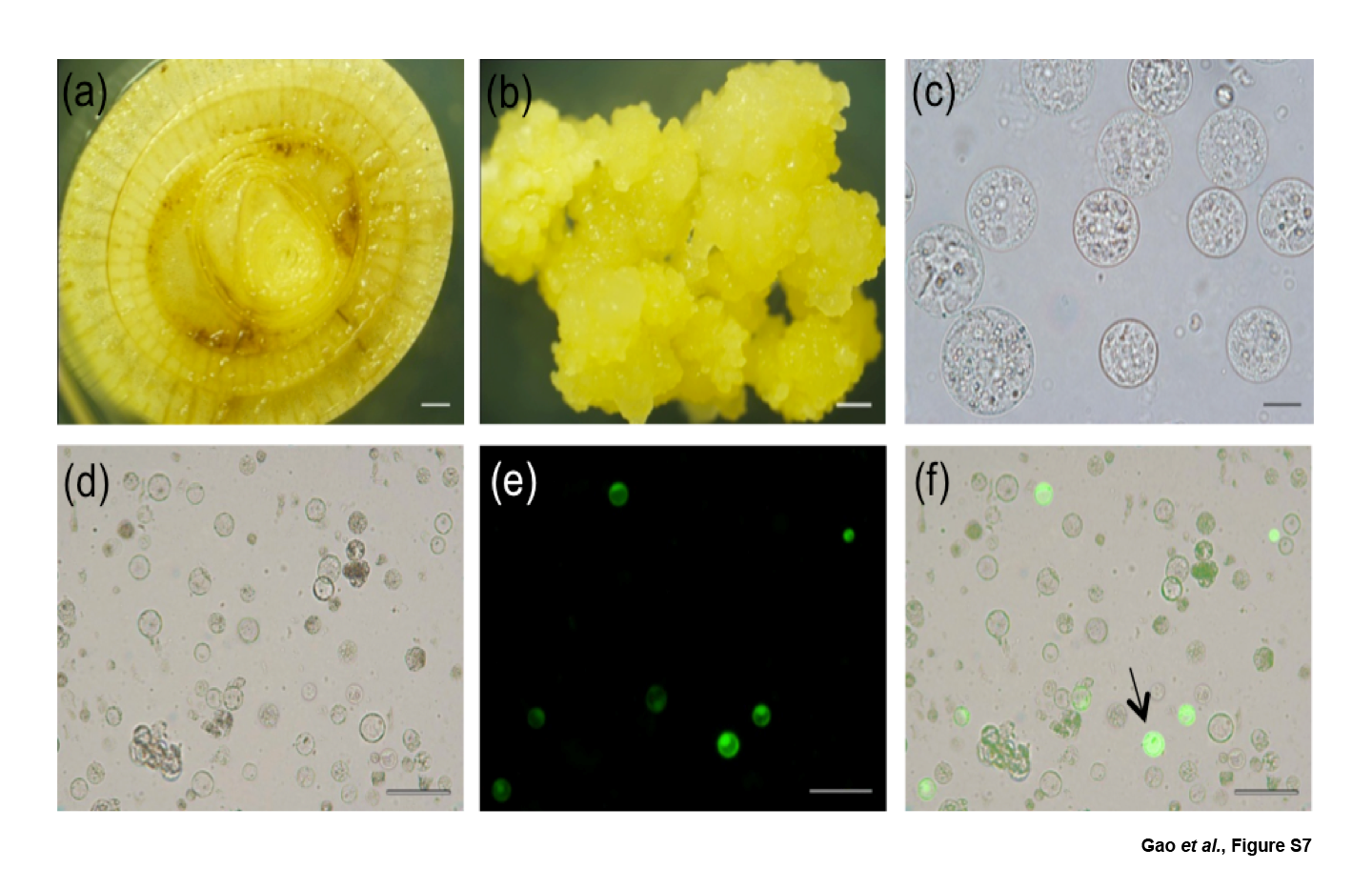

Supplement: Figure S7 — Transient expression of the EYFP reporter gene in sugarcane protoplasts. (a) A seven day-old leaf roll disc growing on MS shoot regeneration medium (9.5x magnification, bar = 1.0 mm); (b) Callus regenerated from leaf roll disc after subculture on MS medium for 4–6 weeks and grown in MS liquid medium to obtain suspension cells (12x magnification, bar = 1.0 mm); (c) Protoplasts isolated from suspension cells under bright light (400x, bar = 20 µm); (d) and (e) Protoplasts transfected with the pUbi:EYFP:Tnos vector expressing EYFP under bright light (100x magnification) and EYFP filter (100x magnification), respectively (bar = 100 µm); (f) Overlaid image of (d) and (e) showing transfection efficiency; a transfected protoplast is indicated by an arrow (bar = 100 µm). Microphotographs of (a) and (b) were collected using an Olympus SZX7 fluorescence microscope with a DP71 camera. Microphotographs of (c), (d), (e) and (f) were obtained with an Olympus BX51 fluorescence microscope with a DP72 camera. (TIF) [file pone.0066046.s007.tif]

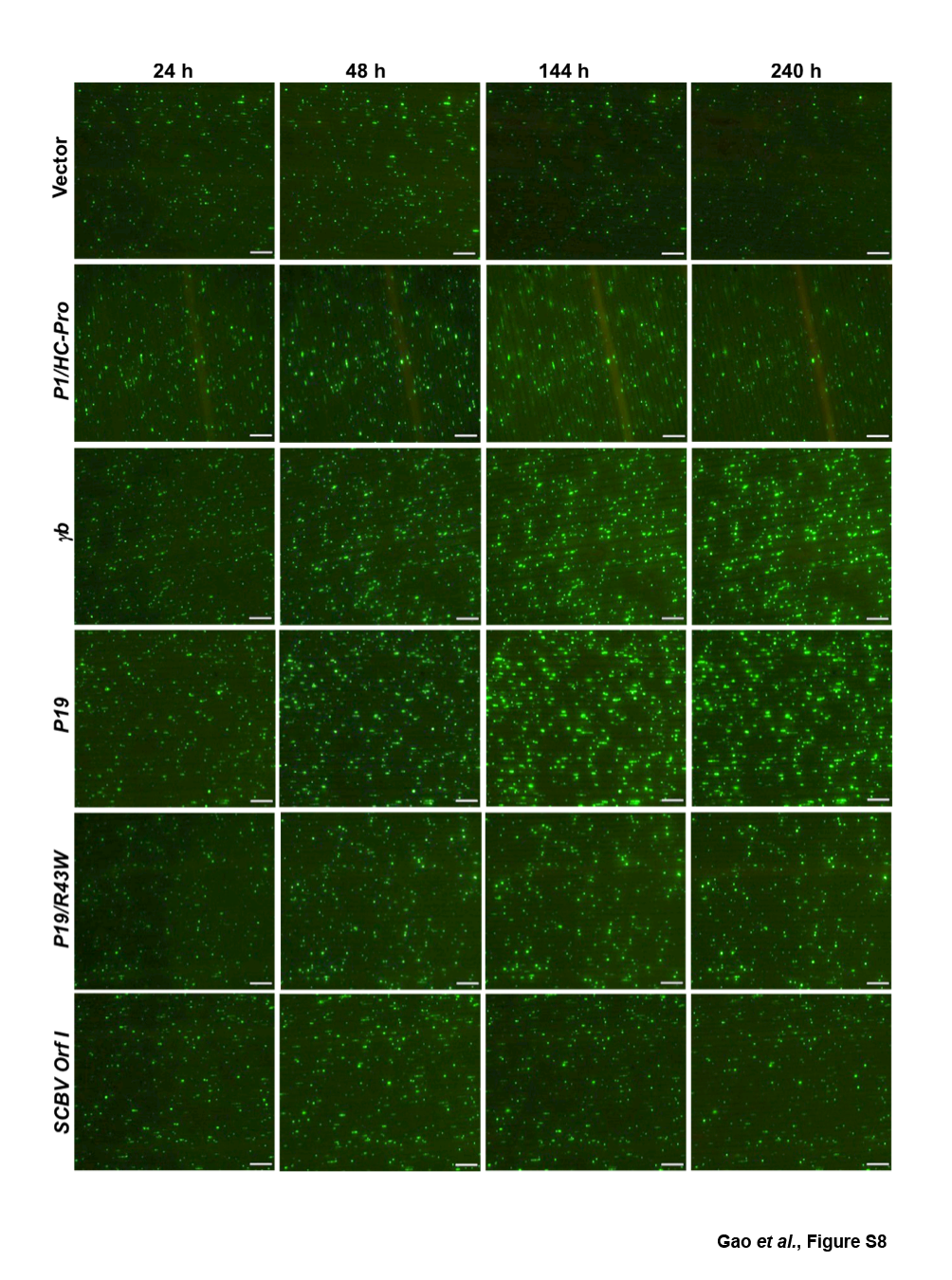

Supplement: Figure S8 — Effect of viral RNA silencing suppressors on transient expression of the EYFP reporter gene in sugarcane young leaf segments. Images of EYFP expression were collected with a SZX7 fluorescence stereomicroscope and a DP71 digital camera (15x magnification) (Olympus) from the same young leaf segments at 24–240 h after co-bombardment with EYFP (pUbi:EYFP:Tnos; Figure S3) (0.25 µg) and each of the RNA silencing suppressors (driven by the Ubi promoter, Figure S3), HC-Pro, γb, P19, P19/R43W and SCBV OrfI (0.5 µg) (bar = 0.5 mm). Vector with no suppressor (pUbi:Tnos; Figure S3) was used as a negative control. (TIF) [file pone.0066046.s008.tif]
