# Supplementary material for: Enhanced Transgene Expression in Sugarcane by Co-Expression of Virus-Encoded RNA Silencing Suppressors
Source: PLoS One. 2013 Jun 14;8(6):e66046. doi: 10.1371/journal.pone.0066046 (PMC3682945; doi:10.1371/journal.pone.0066046)
Supplement: Figure S2 — Suppression of silencing in onion epidermal cells. (a) Example of the expression of the gene encoding the Enhanced Yellow Fluorescent Protein (EYFP) in a single onion cell at two days post-bombardment. At 1–2 h before transformation, onion epidermal peels were prepared under sterile conditions using pointed forceps and placed adaxial side up onto Murashige and Skoog basal salt mixture (MS) media [79] with 0.2 M D-mannitol and 0.2 M D-sorbitol (MS osmoticum). Two explants were used per plate, and each plate was replicated 4–5 times. Genes encoding the suppressors were under control of the maize ubiquitin 1 promoter, as described in Materials and Methods. Plasmid DNA of the appropriate construct was introduced into onion cells using a PDS-1000/He particle delivery system. Bombardment was performed at 9 cm from targets using gold particles (1.0 micron; Bio-Rad Laboratories) coated with plasmids expressing EYFP or viral suppressors under 27 inch Hg and 1100 psi helium pressure. Plasmid DNA was precipitated onto the gold particles using calcium chloride (2.5 M) and spermidine (0.1 M). For co-introduction of two and three different plasmids, 4.5 µg and 3.0 µg of each plasmid was used, respectively. The bombarded epidermal peels were incubated on MS osmoticum for 48–72 h at 25°C in the dark. Fluorescence was monitored using a fluorescence binocular microscope Olympus SZX10 with an excitation wavelength of 490 nm. (b) Comparison of different suppressors. Similarly sized onion peel sections were bombarded as described for (a), and the number of fluorescent cells was counted. (DOC) [file pone.0066046.s002.doc]

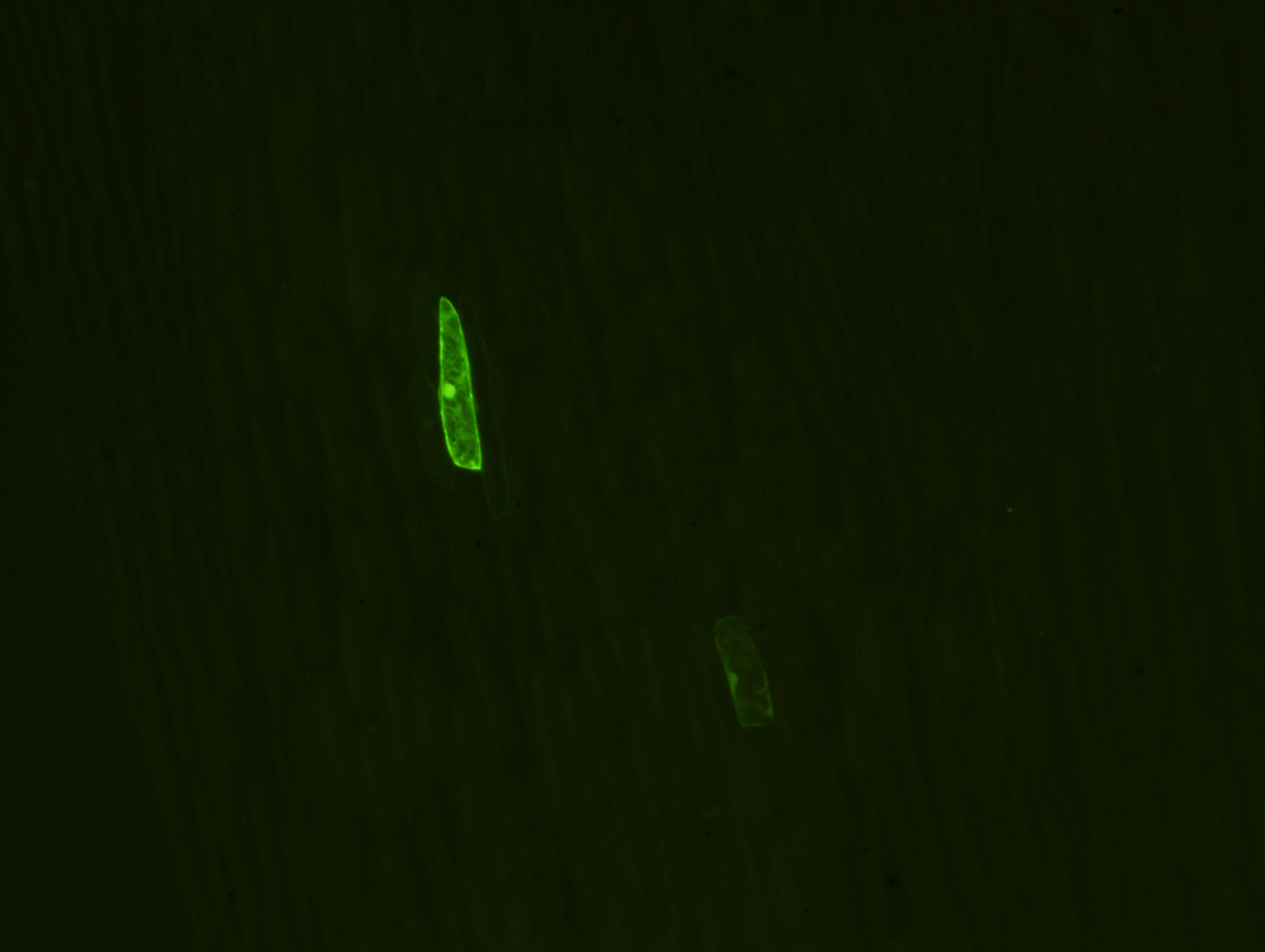
(a)

(b)

| EYFP with suppressor | Fluorescent cell average number |
| --- | --- |
| - | 11.00 |
| P19 | 13.00 |
| P1/HC-Pro | 21.00 |
| γb | 53.00 |
| SCVB OrfI | 117.00 |
| P19 + P1/HC-Pro | 17.00 |
| SCBV OrfI + γb | 59.00 |
| P19 + γb | 53.00 |
| SCVB OrfI + P1/HC-Pro | 67.00 |
